# ZER1 Restrains Pressure Overload‐Induced Cardiac Remodeling by Targeting DVL2 for Gly/N‐Degron‐Dependent Degradation

**DOI:** 10.1002/advs.76308

**Published:** 2026-06-29

**Authors:** Mingchao Jiang, Zhehao Lin, Lu Chen, Ying Ni, Jun Zhou, Wenjuan Zhang, Huilin Wang, Peifeng Ying, Xiu Lu, Kai Wang, Qingran Kong, Naxin Xu, Dingsheng Zhao, Jianwei Li, Guohui Zhong, Xingchen Meng, Sarkawt Hamad, Junmeng Zheng, Yuan Fu, Rongjiang Qin, Xuran Chu, Shangxuan Li, Youyou Li, Yili Wu, Yi Wang, Weihong Song, Yingxian Li, Shukuan Ling

**Affiliations:** ^1^ Oujiang Laboratory (Zhejiang Lab for Regenerative Medicine, Vision and Brain Health) Wenzhou Medical University Wenzhou China; ^2^ Center for Geriatric Medicine Key Laboratory of Alzheimer's Disease of Zhejiang Province The First Affiliated Hospital and Institute of Aging Wenzhou Medical University Wenzhou China; ^3^ State Key Laboratory of Medical Proteomics Beijing Proteome Research Center National Center for Protein Sciences (Beijing) Beijing Institute of Lifeomics Beijing China; ^4^ West China Second University Hospital Sichuan University Chengdu China; ^5^ Frontiers Medical Center Tianfu Jincheng Laboratory Chengdu China; ^6^ Academy of Sports Human Sciences Beijing Sport University Beijing China; ^7^ National Key Laboratory of Space Medicine China Astronaut Research and Training Center Beijing China; ^8^ Center for Physiology and Pathophysiology Institute for Neurophysiology University of Cologne Medical Faculty and University Hospital Cologne Germany; ^9^ Department of Cardiovascular Surgery Sun Yat‐sen Memorial Hospital Sun Yat‐sen University Guangzhou China; ^10^ Department of Physical Education China Agricultural University Beijing China; ^11^ Department of Physical Education Renmin University of China Beijing China

**Keywords:** cardiac hypertrophy, DVL2, heart failure, N‐degron pathway, proteostasis, ZER1

## Abstract

Pathological cardiac hypertrophy drives heart failure progression, but the proteostatic mechanisms restraining maladaptive remodeling remain poorly defined. Here, we identify Zyg‐11‐related regulator 1 (ZER1) as a previously unrecognized Gly/N‐degron proteostatic regulator of pressure overload‐induced remodeling. Failing human hearts and mouse transverse aortic constriction (TAC) hearts show suppression of a CRL2/Gly‐N‐degron signature and reduced ZER1 abundance. Global and cardiomyocyte‐specific *Zer1* loss exacerbates TAC‐induced hypertrophy, fibrosis, and systolic dysfunction. Mechanistically, ZER1 directly binds disheveled segment polarity protein 2 (DVL2) in an N‐terminus‐dependent manner and promotes its K48‐linked polyubiquitination and proteasomal degradation, thereby limiting DVL2 accumulation and downstream CaMKII‐HDAC4‐MEF2C signaling. Cardiomyocyte‐targeted *Dvl2* knockdown abolishes the phenotypic differences between *Zer1* ^fl/fl^ and *Zer1*‐cKO mice after TAC. WWP1 knockdown cannot rescue the phenotype induced by ZER1 deletion, demonstrating that ZER1 is required for *Wwp1* knockdown‐mediated protection against pressure overload‐induced cardiac remodeling. Importantly, cardiomyocyte‐selective AAV9‐mediated restoration of ZER1 after TAC onset attenuates established remodeling and preserves cardiac function. Together, these findings define a ZER1‐DVL2 proteostatic checkpoint that links Gly/N‐degron‐dependent protein quality control to pathological cardiac remodeling and highlights ZER1 as a potential therapeutic target for heart failure.

## Graphical abstract

Pressure overload suppresses cardiomyocyte ZER1, weakening CRL2^Zer1^‐mediated DVL2 degradation and allowing DVL2 accumulation. Elevated DVL2 activates CaMKII‐HDAC4‐MEF2C signaling, drives fetal gene reactivation, and promotes pathological remodeling. Cardiomyocyte‐directed Zer1 restoration enhances K48‐linked DVL2 polyubiquitination and proteasomal turnover, thereby restraining maladaptive signaling and preserving cardiac structure and function.

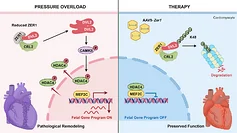

## 1 Introduction

Pressure overload triggers cardiac hypertrophy as an initially adaptive response to hemodynamic stress, yet sustained hypertrophic signaling promotes maladaptive remodeling and progression to heart failure [1, 2]. Despite advances in medical and device‐based therapies, heart failure continues to impose substantial morbidity and mortality, underscoring the need to define new regulatory mechanisms that govern pathological remodeling and identify actionable nodes for intervention [3].

Proteostasis is central to cardiomyocyte homeostasis, and impaired protein quality control accelerates heart failure progression [4, 5]. The ubiquitin‐proteasome system (UPS) is a principal pathway for regulated protein turnover, in which substrate selectivity is dictated largely by E3 ubiquitin ligases [6]. A major logic of substrate recognition is the use of short peptide motifs, or degrons [7]. Among the earliest and best‐characterized degrons are N‐terminal degrons (N‐degrons), which gave rise to the N‐end rule concept linking a protein's N‐terminal residue to its metabolic stability [7, 8, 9]. Subsequent work has expanded N‐degron biology into multiple branches that recognize distinct N‐terminal states and execute selective degradation programs [10, 11, 12, 13, 14, 15, 16, 17, 18]. Genetic perturbation of core N‐degron components disrupts cardiovascular development and compromises myofibrillar organization, supporting the broader premise that N‐degron‐guided ubiquitination contributes to cardiomyocyte proteostasis [19, 20, 21, 22]. However, how specific N‐degron branches operate in adult pathological remodeling remains incompletely defined.

A recently described branch of the N‐degron system‐the Gly/N‐degron pathway‐mediates degradation of proteins bearing small N‐terminal residues, with particular emphasis on N‐terminal glycine [23]. In this pathway, the CRL2 substrate receptors ZYG11B and ZER1 cooperate as substrate‐recognition components of Cullin 2‐RING E3 ligase complexes [24, 25]. Structural studies have shown that ZYG11B and ZER1 use armadillo (ARM) repeats to form an acidic pocket that binds small N‐terminal residues, enabling selective substrate engagement [26]. While these receptors were initially characterized in the context of protein quality control, the Gly/N‐degron pathway has also been linked to regulated signaling events, including control of NLRP1 inflammasome activation after protease cleavage [27]. Whether Gly/N‐degron‐dependent proteostasis participates in cardiac remodeling, and which substrates might connect this pathway to hypertrophic signaling, remains unknown.

Dishevelled (DVL) proteins are central cytoplasmic mediators in both canonical and noncanonical WNT signaling and have established roles in cardiac development and stress remodeling [28, 29, 30, 31]. In cardiomyocytes, DVL2 has been implicated in activating a CaMKII‐HDAC axis that converges on MEF2‐dependent transcriptional reprogramming, a hallmark of pathological hypertrophy [32, 33, 34, 35]. Consistent with the importance of DVL dosage control, DVL proteins are regulated by multiple ubiquitin‐dependent mechanisms, and several E3 ligases and deubiquitinating enzymes have been implicated in shaping DVL stability [36, 37]. Our previous work identified WWP1 as a DVL2‐stabilizing E3 ligase that promotes pressure overload remodeling through atypical K27‐linked ubiquitination [33]. However, whether an opposing ubiquitin‐dependent degradative mechanism restrains DVL2 abundance in the stressed heart remains unknown. Because the DVL2 N terminus contains a putative degron compatible with Gly/N‐degron recognition, this raised the possibility that DVL2 dosage could be controlled not only by stabilizing E3 ligases but also by Gly/N‐degron‐dependent degradation.

Here, we identify ZER1 as a previously unrecognized Gly/N‐degron proteostatic regulator that restrains pressure overload‐induced cardiac remodeling. ZER1 directly recognizes the DVL2 N‐terminus and promotes K48‐linked polyubiquitination and proteasomal degradation of DVL2, thereby limiting DVL2‐CaMKII‐HDAC4‐MEF2C signaling. Using global and cardiomyocyte‐specific *Zer1* loss‐of‐function models, *Dvl2* knockdown epistasis, *Wwp1* knockdown in *Zer1*‐deficient hearts, and therapeutic AAV9‐mediated ZER1 restoration after TAC onset, we establish ZER1‐dependent DVL2 degradation as a cardiomyocyte‐intrinsic proteostatic mechanism and show that the protective effect of *Wwp1* knockdown against pressure overload‐induced cardiac remodeling requires ZER1‐dependent DVL2 proteostatic control.

## 2 Results

### 2.1 CRL2/Gly‐N‐degron Module Activity and ZER1 Expression are Reduced in Hypertrophic and Failing Hearts

To obtain an unbiased view of CRL2/Gly‐N‐degron pathway alterations in human heart failure, we quantified a CRL2 module score based on core CRL2 components and the Gly‐N‐degron substrate receptors (*ZER1*, *ZYG11B*) across a non‐failing (NF) and dilated cardiomyopathy (DCM) RNA‐seq cohort (GSE116250) [38]. The CRL2 module score was lower in DCM hearts than in NF controls (Figure 1A) and inversely associated with a fetal gene program score (*NPPA, NPPB, and MYH7*) across samples (Figure 1B). A similar pattern was observed in an independent mouse pressure‐overload transcriptome dataset (GSE203083) (Figure S1A,B) [39]. Several CRL2 module components showed significant downregulation in either dataset; notably, ZER1 was the only component concordantly downregulated in both human DCM and mouse TAC datasets (Figure S1C).

**FIGURE 1:**
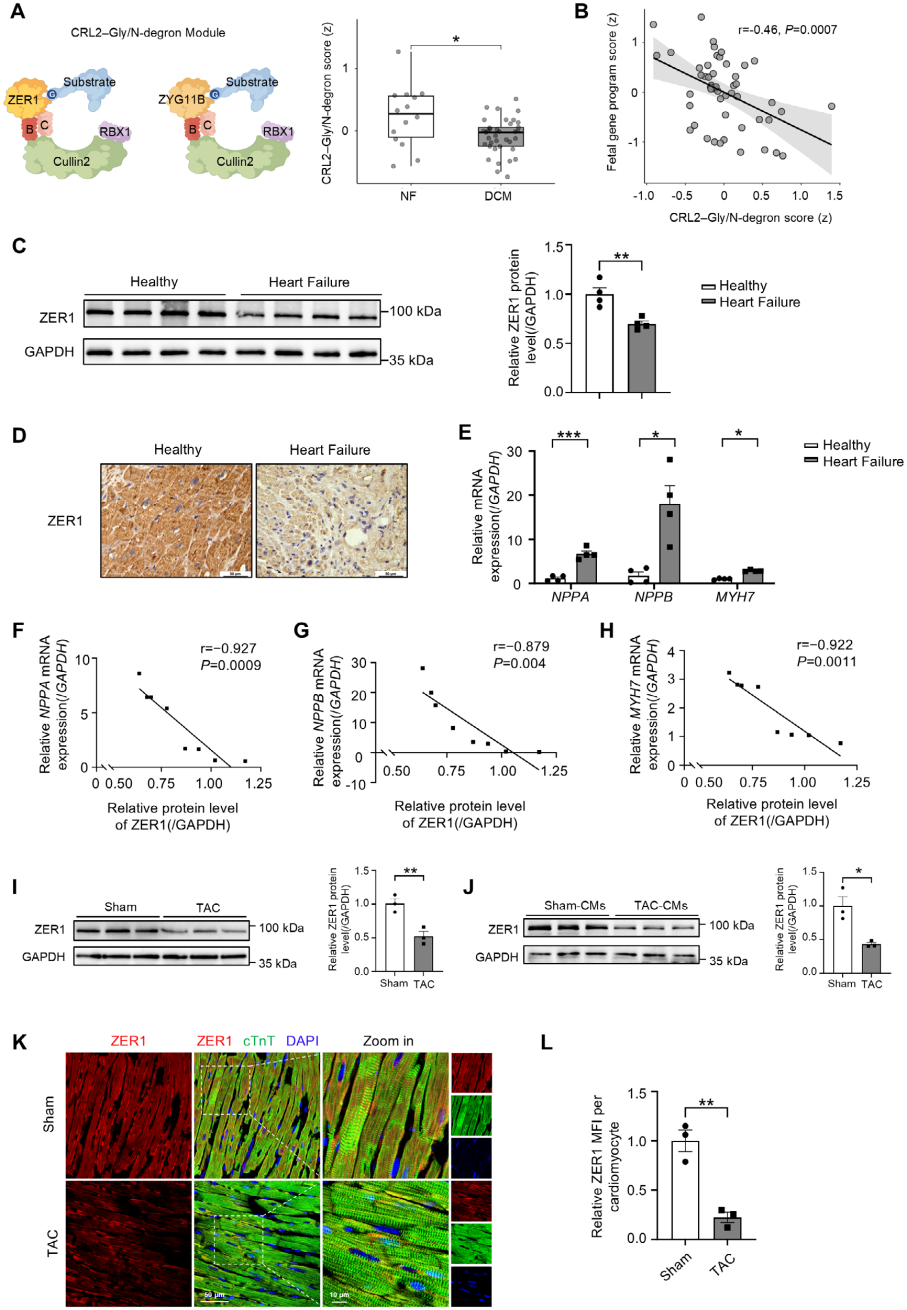
ZER1 is reduced in failing and hypertrophic hearts. (A) Schematic of the CRL2/Gly‐N‐degron module and comparison of CRL2/Gly‐N‐degron module scores between non‐failing (NF) and dilated cardiomyopathy (DCM) human heart samples from GSE116250. (B) Association between CRL2/Gly‐N‐degron module score and fetal gene program score based on *NPPA*, *NPPB*, and *MYH7* expression in human samples from GSE116250. Pearson correlation with two‐sided *p*‐value. (C) Immunoblot analysis and quantification of ZER1 in human myocardial extracts from healthy donors and patients with heart failure (HF) (*n* = 4 per group). (D) Representative ZER1 immunohistochemistry in paraffin‐embedded human heart sections. Scale bar, 50 µm. (E) RT‐qPCR analysis of *NPPA*, *NPPB*, and *MYH7* mRNA levels in human hearts (healthy, *n* = 4; HF, *n* = 4). Values were normalized to *GAPDH*. (F–H) Correlations between myocardial ZER1 protein levels and fetal gene expression (*NPPA*, *NPPB*, and *MYH7*) in human hearts. Pearson correlation with two‐sided *p* value. (I) Immunoblot analysis and quantification of ZER1 in mouse left ventricular lysates 4 weeks after sham or TAC (*n* = 3 per group). (J) Immunoblot analysis and quantification of ZER1 in adult cardiomyocytes isolated 4 weeks after sham or TAC (*n* = 3 per group). (K) Representative immunofluorescence images of mouse heart sections 4 weeks after sham or TAC. ZER1, red; cardiomyocytes (cTnT), green; nuclei (DAPI), blue. Scale bars, 50 and 10 µm. (L) Quantification of ZER1 mean fluorescence intensity per cardiomyocyte in (K) (three fields per heart; *n* = 3 hearts per group). Data are presented as mean ± SEM. Statistical analyses were performed using unpaired two‐tailed Student's *t*‐tests or Mann–Whitney U test for two‐group comparisons. *p < 0.05, **p < 0.01, and ***p < 0.001.

We next assessed ZER1 expression at the protein level in human myocardium. Immunoblotting and immunohistochemistry demonstrated reduced ZER1 abundance in failing human hearts relative to healthy donor controls (Figure 1C,D and Table S1). In the same cohort, *NPPA, NPPB, and MYH7* mRNAs were increased (Figure 1E), and myocardial ZER1 protein abundance negatively associated with fetal gene expression (Figure 1F–H), linking reduced ZER1 to the extent of pathological remodeling.

To determine whether ZER1 is similarly regulated in pressure overload, we analyzed mouse hearts after 4 weeks of transverse aortic constriction (TAC). Immunoblotting and densitometric quantification showed that ZER1 protein was reduced in left ventricular lysates after TAC (Figure 1I). When adult cardiomyocytes were isolated, ZER1 downregulation remained evident in the cardiomyocyte fraction (Figure 1J), whereas ZER1 levels were not detectably altered in isolated non‐cardiomyocytes (Figure S1D). Immunofluorescence staining further showed diminished ZER1 signal within cTnT‐positive cardiomyocytes after TAC (Figure 1K,L). Together, these data identify coordinated suppression of the CRL2/Gly‐N‐degron module in failing and hypertrophic hearts and nominate ZER1 downregulation in cardiomyocytes as a disease‐associated feature of pressure overload remodeling.

### 2.2 Global *Zer1* Deficiency Aggravates Stress‐ and Age‐Associated Cardiac Remodeling

To obtain supportive in vivo evidence for the role of ZER1 in cardiac remodeling, we generated global *Zer1*‐knockout (KO) mice by deleting exons 3–9 of the *Zer1* locus. Successful *Zer1* deletion was confirmed by genotyping and by loss of cardiac ZER1 protein (Figure S2A–C). When subjected to TAC for 4 weeks, *Zer1* KO mice developed more severe cardiac hypertrophy, fibrosis, fetal gene reactivation, and LV systolic dysfunction than WT controls (Figures S3A–H and S4A–L). In addition, aged *Zer1* KO mice showed spontaneous cardiac remodeling under basal conditions, with evidence of cardiac hypertrophy and LV dysfunction at 8 months of age (Figure S5A–M). Together, these supportive data indicate that ZER1 loss sensitizes the heart to stress‐ and age‐associated remodeling.

### 2.3 Cardiac IP‐MS Identifies DVL2 as an N‐Terminus‐Dependent ZER1 Interactor

To identify ZER1‐associated proteins in a cardiac context, we performed endogenous ZER1 immunoprecipitation followed by mass spectrometry (IP‐MS) using mouse left ventricular lysates (Figure 2A). This analysis identified 53 significantly enriched ZER1‐associated proteins relative to IgG controls. To minimize post hoc selection bias, candidates were prioritized using a pre‐defined Gly/N‐degron‐guided workflow, consisting of an N‐terminus compatibility filter followed by GO‐BP‐based tiering for remodeling relevance (Figure S6A and Table S2). Within this framework, DVL2 emerged as a high‐priority Tier 1 candidate with a Gly/N‐degron‐compatible N terminus and ranked among the top Tier 1 candidates by IP‐MS enrichment (Figure S6B and Table S3). Targeted endogenous Co‐IP from mouse heart lysates further supported selective ZER1‐DVL2 association compared with two closely ranked Tier 1 candidates, including CaMKII and EZR (Figure S6C). In addition, DVL1 and DVL3 did not show comparable enrichment under the same conditions, supporting specificity within the DVL family (Figure S6D).

**FIGURE 2:**
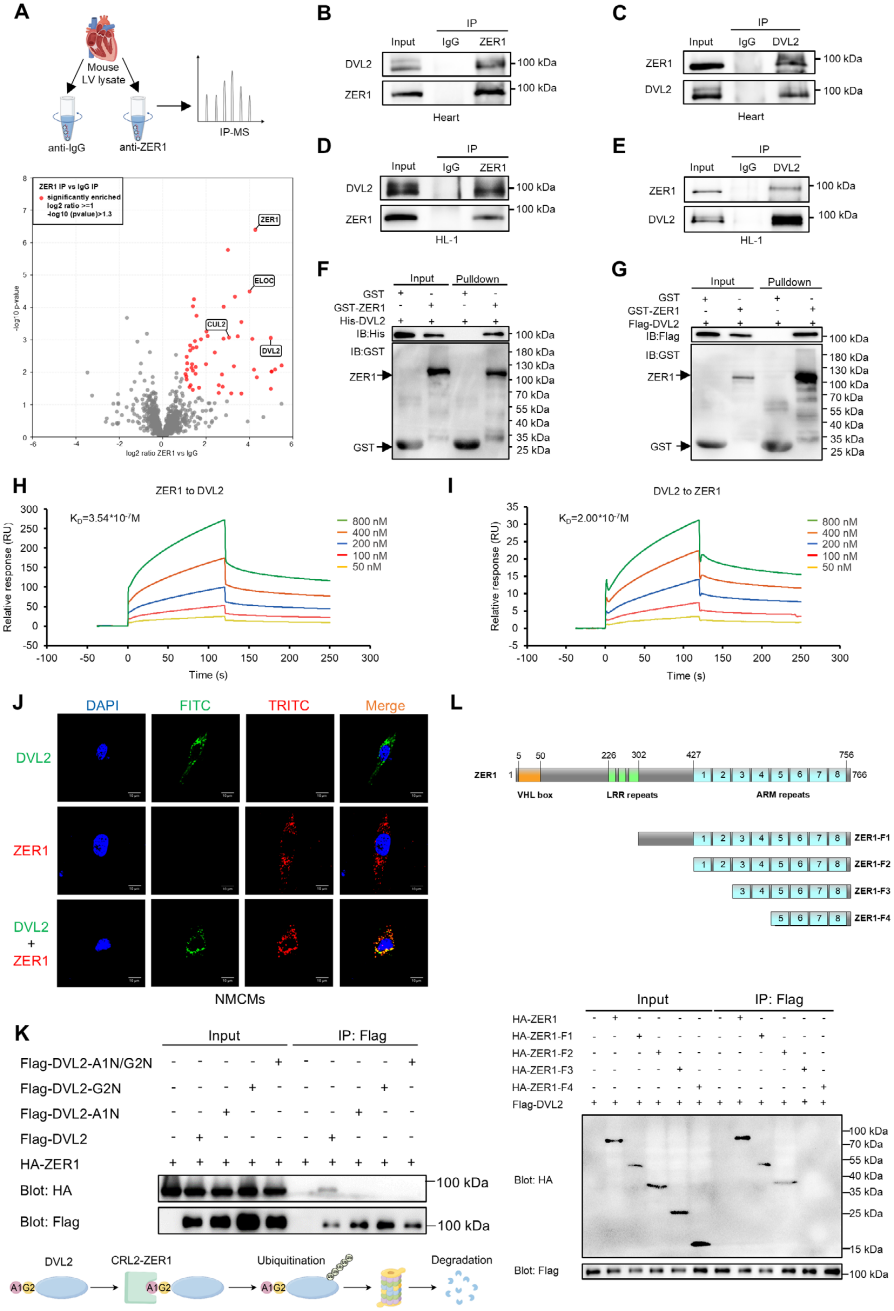
Cardiac IP‐MS identifies DVL2 as an N‐terminus‐dependent ZER1 interactor. (A) Endogenous ZER1 immunoprecipitation followed by mass spectrometry (IP‐MS) using mouse left ventricular lysates. Volcano plot comparing ZER1 immunoprecipitation with IgG control. Significantly enriched proteins are shown in red. ELOC, Elongin C; CUL2, Cullin 2. (B, C) Reciprocal endogenous Co‐IP of ZER1 (B) and DVL2 (C) from WT mouse heart lysates. (D, E) Reciprocal endogenous Co‐IP of ZER1 (D) and DVL2 (E) in HL‐1 cardiomyocytes. (F) GST pull‐down showing direct interaction between GST‐ZER1 and His‐DVL2. (G) GST pull‐down showing interaction between bacterially expressed GST‐ZER1 and Flag‐DVL2 from transfected HEK293T cell lysates. (H, I) SPR analysis of ZER1‐DVL2 binding. GST‐ZER1 immobilized with His‐DVL2 as analyte (H), or His‐DVL2 immobilized with GST‐ZER1 as analyte (I). Dissociation constants (K_D_) are indicated. (J) Confocal microscopy showing co‐localization of endogenous ZER1 and DVL2 in neonatal mouse cardiomyocytes. Scale bar, 10 µm. (K) Co‐IP showing that mutation of DVL2 Ala1 and/or Gly2 to Asn (A1N, G2N, or A1N/G2N) abolishes ZER1‐DVL2 association in HEK293T cells. (L) Domain mapping of ZER1 required for DVL2 binding using ZER1 truncation constructs.

We next validated the ZER1‐DVL2 interaction in cardiac‐relevant systems. Reciprocal endogenous co‐immunoprecipitation from WT mouse heart lysates confirmed that ZER1 and DVL2 associate in vivo (Figure 2B,C). This interaction was recapitulated at the endogenous level in HL‐1 cardiomyocytes (Figure 2D,E). In HEK293T cells, endogenous and semi‐endogenous Co‐IP assays further supported robust association between ZER1 and DVL2 (Figure S7A–D).

To determine whether the interaction is direct, we performed in vitro binding assays using purified proteins. GST pull‐down assays demonstrated direct binding between GST‐ZER1 and His‐DVL2 (Figure 2F). In an independent configuration, bacterially expressed GST‐ZER1 efficiently captured Flag‐DVL2 from transfected HEK293T lysates (Figure 2G). Surface plasmon resonance (SPR) measurements quantified this interaction and revealed submicromolar affinity between ZER1 and DVL2 (Figure 2H,I). In neonatal mouse cardiomyocytes (NMCMs), confocal microscopy showed cytoplasmic co‐localization of ZER1 and DVL2 (Figure 2J), which was also observed in HEK293T cells (Figure S7E).

ZER1 has been shown to recognize Gly/N‐degrons through its ARM‐repeat domain [23, 24, 26]. We therefore tested whether the extreme N‐terminus of DVL2 is required for ZER1 recognition. Substituting Ala1 and/or Gly2 with Asn (A1N, G2N, or A1N/G2N) abolished DVL2 association with ZER1 in Co‐IP assays (Figure 2K), indicating that both the identity and local context of the first two N‐terminal residues are required for DVL2 binding. Structural prediction using AlphaFold 3 suggested that ZER1 engages the DVL2 N‐terminus within an acidic pocket formed by the ARM repeats (Figure S7F,G). Consistently, SPR using synthetic DVL2 N‐terminal peptides demonstrated strong binding to the WT peptide, whereas A1N, G2N, or A1N/G2N mutations markedly weakened the interaction (Figure S7H–K).

Finally, to define the ZER1 domain requirements for DVL2 binding, we mapped the interaction using ZER1 truncation constructs spanning distinct structural modules. ZER1 contains an N‐terminal VHL motif, central LRRs, and C‐terminal ARM repeats; Co‐IP mapping showed that constructs retaining ARM1‐ARM8 bound DVL2, whereas fragments containing only subsets of ARM repeats failed to interact (Figure 2L), indicating that the full ARM‐repeat region is required for DVL2 recognition in cells. Together, these data establish DVL2 as a cardiac ZER1 interactor whose recognition depends on its N‐terminal degron.

### 2.4 ZER1 Drives Proteasomal Degradation of DVL2

We next asked whether ZER1 regulates DVL2 abundance. In HEK293T cells, *ZER1* overexpression reduced DVL2 protein levels (Figure 3A and Figure S8A), whereas ZER1 knockdown increased endogenous DVL2 abundance (Figure 3B and Figure S8B). To determine whether this effect reflects altered protein stability, we performed cycloheximide (CHX) chase assays. Co‐expression of ZER1 accelerated DVL2 turnover (Figure 3C), whereas ZER1 silencing prolonged the DVL2 half‐life (Figure 3D). *DVL2* mRNA levels were not detectably altered by *ZER1* overexpression or knockdown (Figure S8C,D), supporting post‐transcriptional regulation.

**FIGURE 3:**
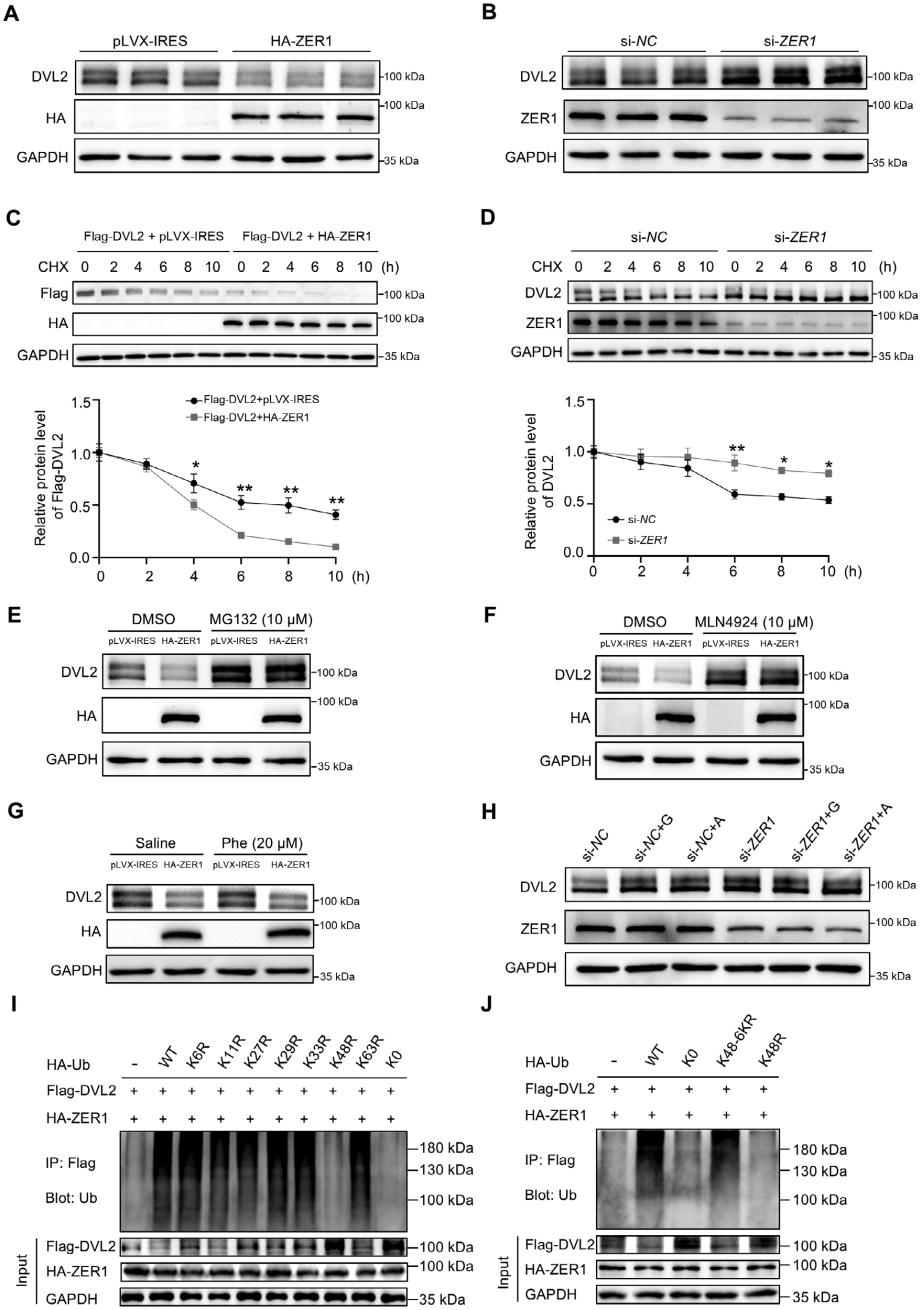
ZER1 drives proteasomal degradation of DVL2. (A) Immunoblot analysis of DVL2 following HA‐ZER1 overexpression in HEK293T cells. (B) Immunoblot analysis of endogenous DVL2 following ZER1 knockdown in HEK293T cells. (C, D) Cycloheximide (CHX) chase assays showing altered DVL2 stability with ZER1 overexpression (C) or ZER1 knockdown (D). (E–G) Effects of MG132 (10 µm) (E), MLN4924 (10 µm) (F), or phenylalanine (Phe, 20 µm) (G) on ZER1‐mediated DVL2 reduction in HEK293T cells. (H) Effects of glycine or alanine supplementation on DVL2 abundance and ZER1‐dependent DVL2 reduction. (I, J) Ubiquitination assays using ubiquitin lysine‐to‐arginine (KR) mutants (I) or lysine‐only (K‐only) mutants (J) to define linkage requirements for ZER1‐driven DVL2 polyubiquitination. Data are presented as mean ± SEM. Statistical analyses were performed using two‐way ANOVA followed by Holm‐Šídák's post hoc test. *p < 0.05 and **p < 0.01.

We then tested whether ZER1‐mediated loss of DVL2 depends on the ubiquitin‐proteasome system and CRL activity. Proteasome inhibition with MG132 blocked ZER1‐driven DVL2 reduction (Figure 3E and Figure S8E,F). Inhibition of cullin neddylation with MLN4924 similarly stabilized DVL2 to a degree comparable to MG132 (Figure 3F and Figure S8G,H), consistent with a Cullin‐RING ligase‐dependent mechanism [40]. In contrast, phenylalanine (Phe) treatment did not stabilize DVL2 (Figure 3G and Figure S8I,J), arguing against involvement of the UBR‐type N‐recognin system [27, 41]. Consistent with N‐terminal residue‐dependent regulation, glycine or alanine supplementation increased DVL2 protein abundance and attenuated ZER1‐dependent DVL2 degradation (Figure 3H and Figure S8K) [41, 42, 43].

Finally, we examined the ubiquitin linkage underlying ZER1‐dependent DVL2 turnover. Using lysine‐to‐arginine (KR) ubiquitin mutants, ZER1‐driven DVL2 polyubiquitination was markedly reduced when K48 was unavailable (Figure 3I). Complementary experiments with lysine‐only (K‐only) ubiquitin mutants showed that K48‐only ubiquitin supported robust DVL2 polyubiquitination in the presence of ZER1 (Figure 3J). To confirm this regulatory relationship in cardiomyocytes, we performed CHX chase assays in NMCMs isolated from WT and *Zer1*‐KO mice. DVL2 turnover was delayed in *Zer1*‐deficient NMCMs, supporting a cardiomyocyte‐autonomous role for ZER1 in DVL2 degradation (Figure S8L). Together, these data indicate that ZER1 promotes CRL‐ and proteasome‐dependent turnover of DVL2 via K48‐linked polyubiquitination.

### 2.5 ZER1 Restrains DVL2‐CaMKII‐HDAC4‐MEF2C Signaling

We next tested whether ZER1‐DVL2 engagement modulates hypertrophic signaling, focusing on the CaMKII‐HDAC4 axis and MEF2C transcriptional output. In HEK293T cells, *ZER1* overexpression reduced DVL2 abundance and decreased phosphorylation of CaMKII (Thr287) and HDAC4 (Ser246) (Figure 4A and Figure S9A). Conversely, ZER1 silencing increased DVL2 and enhanced CaMKII/HDAC4 phosphorylation (Figure 4B and Figure S9B). Simultaneous DVL2 knockdown abolished the pathway activation induced by ZER1 depletion (Figure 4C and Figure S9C–F), placing DVL2 downstream of ZER1 in controlling CaMKII‐HDAC4 signaling.

**FIGURE 4:**
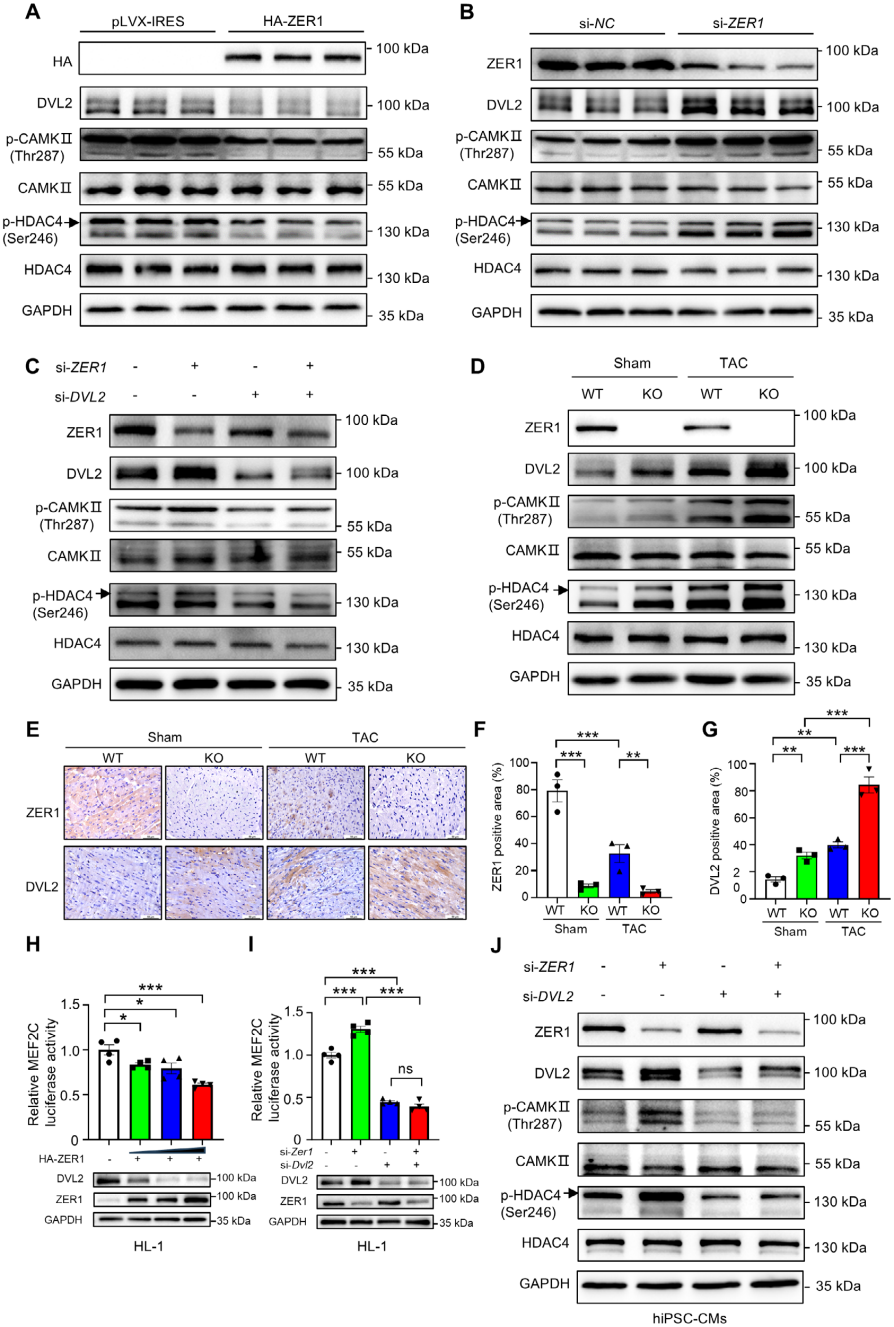
ZER1 restrains DVL2‐CaMKII‐HDAC4‐MEF2C signaling. (A–C) Immunoblot analysis of DVL2‐CaMKII‐HDAC4 signaling following HA‐ZER1 overexpression (A), *ZER1* knockdown (B), or combined *ZER1* and *DVL2* knockdown (C) in HEK293T cells. (D) Immunoblot analysis of the ZER1‐DVL2‐CaMKII‐HDAC4 axis in WT and *Zer1* KO mouse hearts after sham or TAC. (E–G) Immunohistochemistry and quantification of ZER1 and DVL2 in WT and Zer1 KO hearts after sham or TAC. Scale bar, 50 µm. (H, I) MEF2C‐dependent luciferase reporter assays in HL‐1 cells following HA‐ZER1 overexpression (H) or *Zer1* silencing with or without *Dvl2* knockdown (I). (J) Immunoblot analysis of DVL2‐CaMKII‐HDAC4 signaling in hiPSC‐derived cardiomyocytes following *ZER1* knockdown, *DVL2* knockdown, or combined knockdown. Data are presented as mean ± SEM. Statistical analyses were performed using one‐way or two‐way ANOVA followed by Holm–Šídák's post hoc test. ns, not significant; *p < 0.05, **p < 0.01, and ***p < 0.001.

We next evaluated this axis in vivo. In cardiac lysates from WT and *Zer1* KO mice subjected to 4 weeks of TAC, *Zer1* deficiency was associated with increased DVL2 abundance and enhanced phosphorylation of CaMKII and HDAC4 (Figure 4D and Figure S9G–J). Immunohistochemistry in TAC hearts further supported an inverse relationship between ZER1 and DVL2 in situ (Figure 4E). Quantification of IHC staining confirmed reduced ZER1‐positive area and increased DVL2‐positive area in Zer1 KO hearts after TAC (Figure 4F,G).

To link these signaling changes to transcriptional output, we assessed MEF2C activity. In HL‐1 cardiomyocytes, *ZER1* overexpression suppressed MEF2C‐dependent reporter activity (Figure 4H). Conversely, Zer1 silencing increased MEF2C activity, and this increase was largely abolished by concomitant Dvl2 knockdown (Figure 4I), supporting DVL2‐dependent regulation of MEF2C downstream of ZER1.

Finally, we tested whether this pathway is conserved in a human cardiomyocyte setting. In hiPSC‐derived cardiomyocytes (hiPSC‐CMs) with validated cardiomyocyte identity (Figure S9K–L), ZER1 knockdown increased DVL2 and enhanced CaMKII/HDAC4 phosphorylation, while co‐silencing DVL2 mitigated these effects (Figure 4J and Figure S9M–P). Because DVL2 participates in WNT signaling, we also examined representative canonical WNT pathway markers in *Zer1*‐KO hearts after TAC (Figure S10A–D). These analyses did not reveal broad activation of canonical WNT signaling, suggesting that ZER1 loss preferentially amplifies the DVL2‐CaMKII‐HDAC4‐MEF2C axis in this pressure‐overload setting. Together, these data support a model in which ZER1 limits DVL2 accumulation to restrain CaMKII‐HDAC4 activation and downstream MEF2C signaling.

### 2.6 Cardiomyocyte‐Specific *Zer1* Deletion Exacerbates Pressure Overload Remodeling via DVL2

Because ZER1 is expressed across multiple cardiac cell types, we tested whether the in vivo phenotype reflects a cardiomyocyte‐intrinsic function. We generated cardiomyocyte‐specific Zer1 knockout mice (*Zer1*‐cKO) and confirmed efficient and tissue‐selective deletion (Figure S11A–E). To further assess the cardiomyocyte‐autonomous consequence of *Zer1* loss, we isolated NMCMs from *Zer1* ^fl/fl^ and Zer1‐cKO mice and challenged them with AngII (Figure S11F). *Zer1*‐deficient NMCMs exhibited an exaggerated hypertrophic response compared with *Zer1* ^fl/fl^ NMCMs (Figure S11G,H). After sham surgery, *Zer1*‐cKO mice did not show overt baseline abnormalities. Following TAC, however, cardiomyocyte‐specific deletion recapitulated adverse remodeling, with increased cardiac hypertrophy and fibrosis and reduced systolic function compared with *Zer1* ^fl/fl^ controls (Figure 5A–F).

**FIGURE 5:**
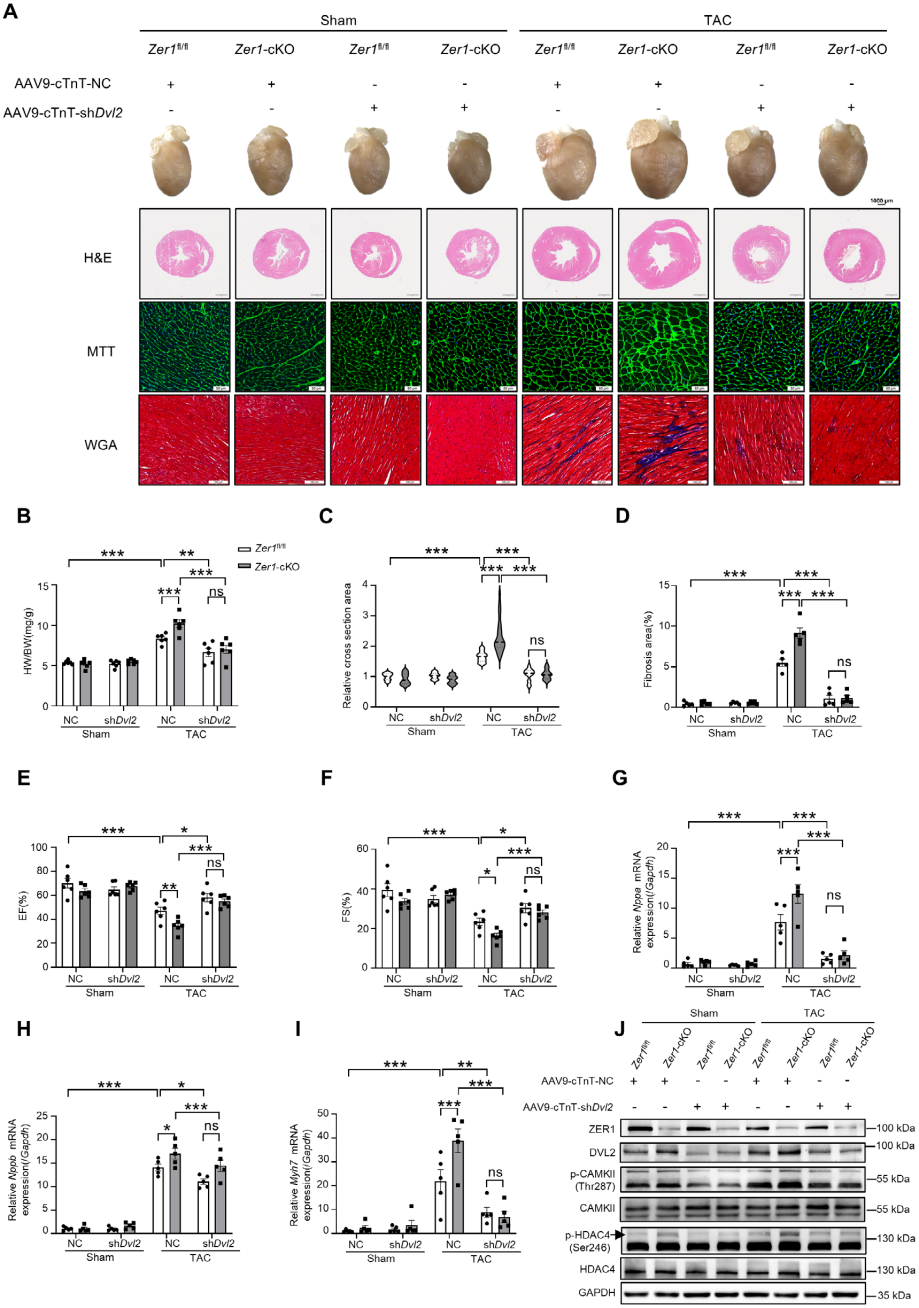
Cardiomyocyte‐specific *Zer1* deletion exacerbates pressure overload remodeling via DVL2. (A) Representative gross hearts and histological analyses from *Zer1* ^fl/fl^ and *Zer1*‐cKO mice subjected to sham or TAC, with AAV9‐cTnT‐NC or AAV9‐cTnT‐sh*Dvl2*. H&E, MTT, and WGA staining are shown. Scale bars, 1 mm for H&E and 50 µm for MTT and WGA. (B) HW/BW ratios (*n* = 6 mice per group). (C) Quantification of cardiomyocyte CSA by WGA staining (*n* = 5 hearts per group; ten fields per heart). (D) Quantification of myocardial fibrosis (*n* = 5 hearts per group). (E, F) Echocardiographic evaluation of EF and FS (*n* = 6 mice per group). (G–I) RT‐qPCR analysis of *Nppa*, *Nppb*, and *Myh7*; values were normalized to *Gapdh* (*n* = 5 mice per group). (J) Immunoblot analyses of ZER1, DVL2, and CaMKII‐HDAC4 signaling in heart lysates from the indicated groups. Data are presented as mean ± SEM. Statistical analyses were performed using two‐way ANOVA followed by Holm–Šídák's post hoc test. ns, not significant; *p < 0.05, **p < 0.01, and ***p < 0.001.

To directly test whether DVL2 accumulation mediates the pathological consequences of ZER1 loss, we performed cardiomyocyte‐targeted Dvl2 knockdown using AAV9‐cTnT‐sh*Dvl2* in *Zer1* ^fl/fl^ and *Zer1*‐cKO mice subjected to TAC (Figure S12A,B). Robust myocardial mCherry fluorescence confirmed efficient AAV9‐cTnT‐sh*Dvl2* delivery, and *Dvl2* mRNA was markedly reduced in treated hearts (Figure S12C,D). Dvl2 knockdown reduced hypertrophic growth and fibrosis (Figure 5A–D) and improved systolic function as assessed by echocardiography (Figure 5E,F and Figure S12E,N). TAC‐induced fetal gene activation was also blunted by Dvl2 knockdown (Figure 5G–I). Immunoblotting confirmed effective reduction of DVL2 and normalization of downstream CaMKII‐HDAC4 activation in treated hearts (Figure 5J and Figure S12O–R). Notably, following TAC and cardiomyocyte‐specific Dvl2 knockdown, differences in hypertrophic indices and systolic function between *Zer1* ^fl/fl^ and *Zer1*‐cKO mice were no longer detectable, supporting an essential role for DVL2 in mediating the pathological effects of ZER1 loss under pressure overload.

### 2.7 ZER1 is Required for *Wwp1* Knockdown‐Mediated Protection Against Pressure Overload‐Induced Cardiac Remodeling

Because ZER1 promotes DVL2 degradation whereas WWP1 has been reported to stabilize DVL2, we next asked how these opposing E3‐dependent mechanisms are functionally related during pressure overload remodeling. Specifically, we tested whether reducing the WWP1‐mediated stabilizing input could compensate for loss of ZER1‐dependent DVL2 degradation. To this end, WT and *Zer1* KO mice were subjected to TAC and treated with AAV9‐cTnT‐sh*Wwp1* or control virus (Figure 6A,B). Efficient cardiac delivery and *Wwp1* knockdown were confirmed by EGFP fluorescence, reduced *Wwp1* mRNA, and reduced WWP1 protein abundance (Figure S13A–C).

**FIGURE 6:**
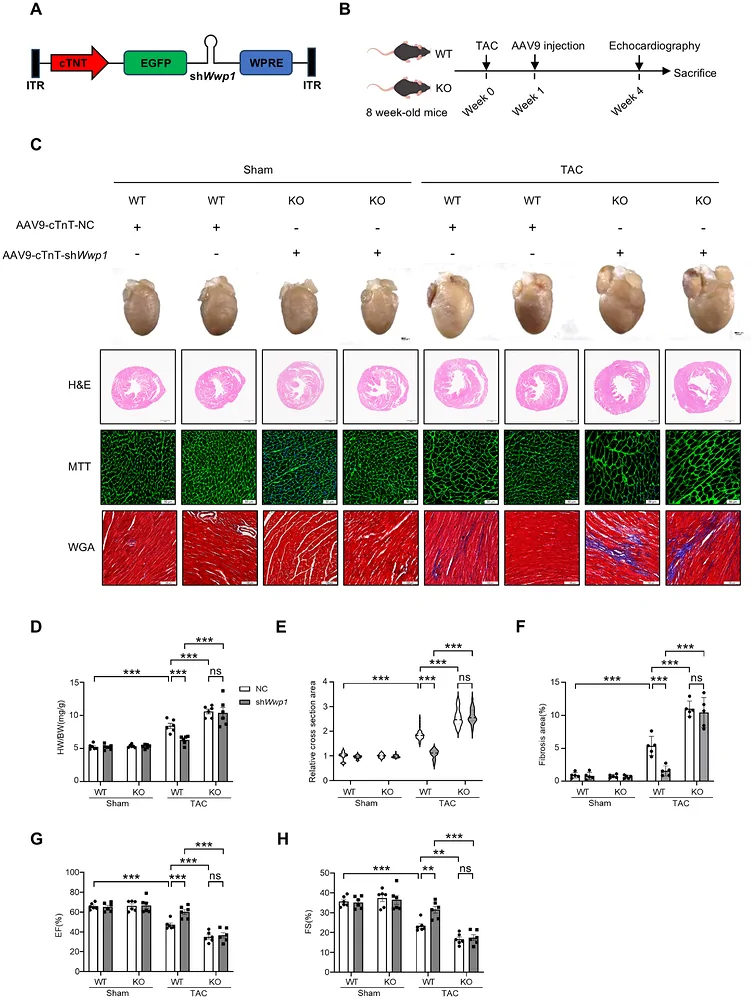
ZER1 is required for *Wwp1* knockdown‐mediated protection against pressure overload‐induced cardiac remodeling. (A) Schematic of the AAV9‐cTnT‐sh*Wwp1* vector. (B) Experimental timeline for AAV9 delivery in WT and *Zer1* KO mice subjected to TAC. (C) Representative gross hearts and histological analyses from WT and *Zer1* KO mice subjected to sham or TAC, with AAV9‐cTnT‐NC or AAV9‐cTnT‐sh*Wwp1*. H&E, WGA, and Masson's trichrome staining are shown. Scale bars, 1 mm for H&E and 50 µm for MTT and WGA. (D) HW/BW ratios (*n* = 6 mice per group). (E) Quantification of cardiomyocyte cross‐sectional area by WGA staining (*n* = 5 hearts per group; ten fields per heart). (F) Quantification of myocardial fibrosis (*n* = 5 hearts per group). (G, H) Echocardiographic evaluation of EF and FS (*n* = 6 mice per group). Data are presented as mean ± SEM. Statistical analyses were performed using two‐way ANOVA followed by Holm–Šídák's post hoc test. ns, not significant; *p < 0.05, **p < 0.01 and ***p < 0.001.

In WT mice after TAC, *Wwp1* knockdown attenuated cardiac hypertrophy and fibrosis and improved LV systolic function, as shown by reduced HW/BW ratio, smaller cardiomyocyte cross‐sectional area (CSA), decreased fibrotic area, and improved EF and FS (Figure 6C–H and Figure S13D–M). However, these protective effects were largely abolished in *Zer1*‐KO mice, in which *Wwp1* knockdown failed to further reduce hypertrophy, fibrosis, or cardiac dysfunction after TAC (Figure 6C–H). These findings indicate that ZER1 is required for *Wwp1* knockdown‐mediated protection against pressure overload‐induced cardiac remodeling.

### 2.8 Therapeutic Cardiomyocyte‐Specific ZER1 Restoration Attenuates Pressure Overload Remodeling

To evaluate whether ZER1 restoration can therapeutically reverse established pressure overload remodeling, we delivered AAV9‐cTnT‐*Zer1* or control vector 1 week after TAC and assessed cardiac remodeling 3 weeks later (Figure 7A). Efficient cardiomyocyte‐targeted transduction was verified by GdGreen reporter expression in the heart (Figure S14A).

**FIGURE 7:**
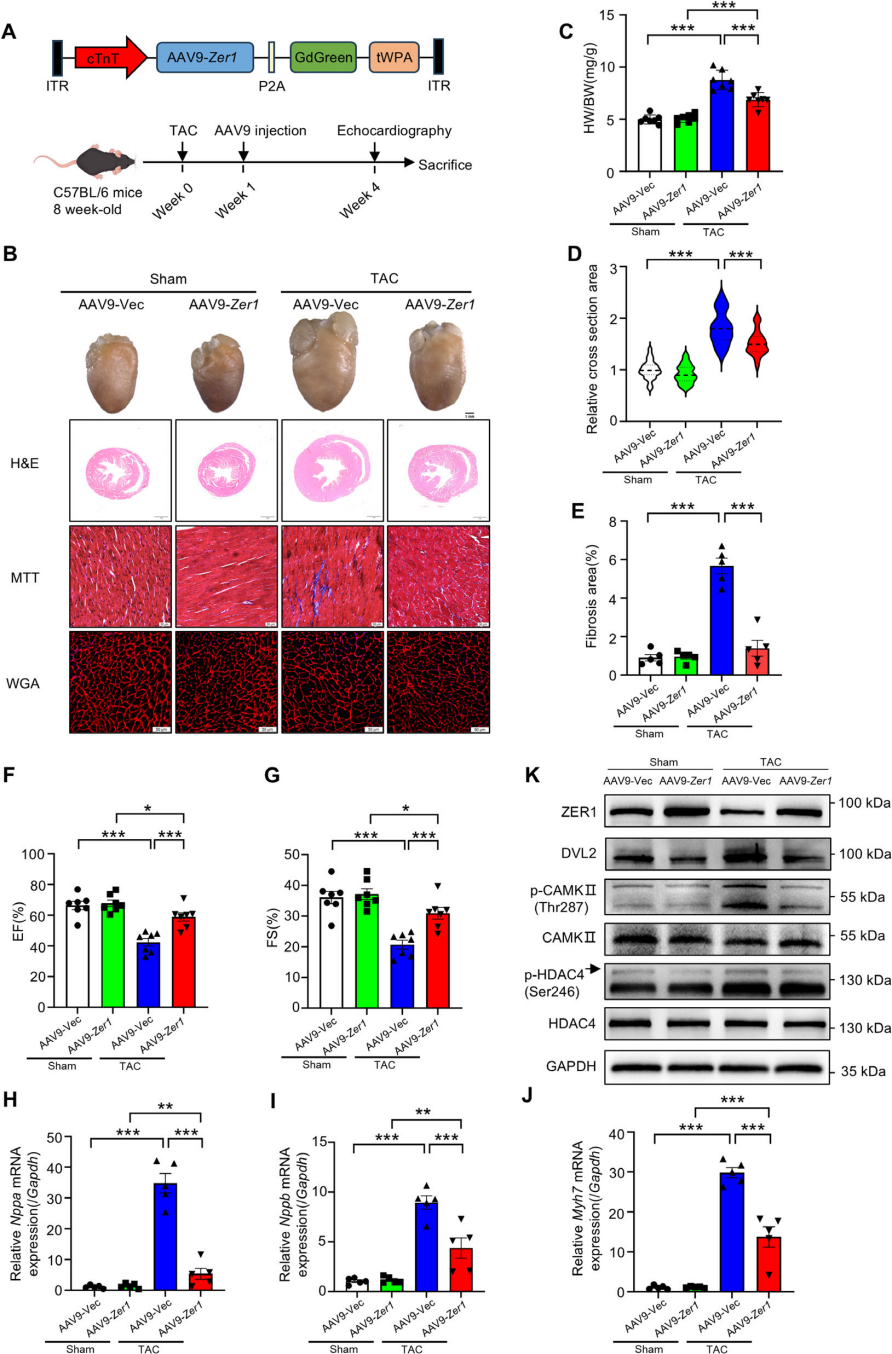
Therapeutic cardiomyocyte‐specific ZER1 expression attenuates pressure overload remodeling. (A) AAV9‐cTnT‐*Zer1* construct and experimental timeline for therapeutic ZER1 expression after TAC. (B) Representative gross hearts and histological analyses 4 weeks after sham or TAC. H&E, WGA, and Masson's trichrome staining are shown. Scale bars, 1 mm for H&E and 50 µm for WGA and Masson's trichrome. (C) HW/BW ratios (*n* = 7 mice per group). (D) Quantification of cardiomyocyte cross‐sectional area by WGA staining (*n* = 5 hearts per group; ten fields per heart). (E) Quantification of myocardial fibrosis (*n* = 5 hearts per group). (F, G) Echocardiographic evaluation of EF and FS (*n* = 7 mice per group). (H–J) RT‐qPCR analysis of *Nppa*, *Nppb*, and *Myh7* mRNA levels; values were normalized to *Gapdh* (*n* = 5 mice per group). (K) Immunoblot analyses of ZER1, DVL2, and CaMKII/HDAC4 phosphorylation in hearts with the indicated treatments. Data are presented as mean ± SEM. Statistical analyses were performed using two‐way ANOVA followed by Holm–Šídák's post hoc test. *p < 0.05, **p < 0.01, and ***p < 0.001.

At 4 weeks after TAC, histological analysis showed that AAV9‐cTnT‐*Zer1* reduced hypertrophic remodeling and diminished myocardial fibrosis compared with vector controls (Figure 7B). Consistently, HW/BW ratios were lower in the AAV9‐cTnT‐*Zer1* group, cardiomyocyte CSA was reduced, and myocardial fibrosis was decreased (Figure 7C–E).

Echocardiographic analysis demonstrated improved systolic function in AAV9‐cTnT‐*Zer1*‐treated mice, with higher EF and FS compared with vector controls (Figure 7F–G and Figure S14B–K).

At the molecular level, AAV9‐cTnT‐*Zer1* suppressed TAC‐induced fetal gene activation, as indicated by reduced *Nppa, Nppb*, and *Myh7* expression (Figure 7H–J). Immunoblotting confirmed increased ZER1 abundance and reduced DVL2 protein levels, accompanied by decreased phosphorylation of CaMKII and HDAC4 in TAC hearts (Figure 7K and Figure S14L–O). Together, these data indicate that cardiomyocyte‐targeted ZER1 restoration after pressure overload onset is sufficient to attenuate maladaptive remodeling and preserve cardiac function (Figure 8).

**FIGURE 8:**
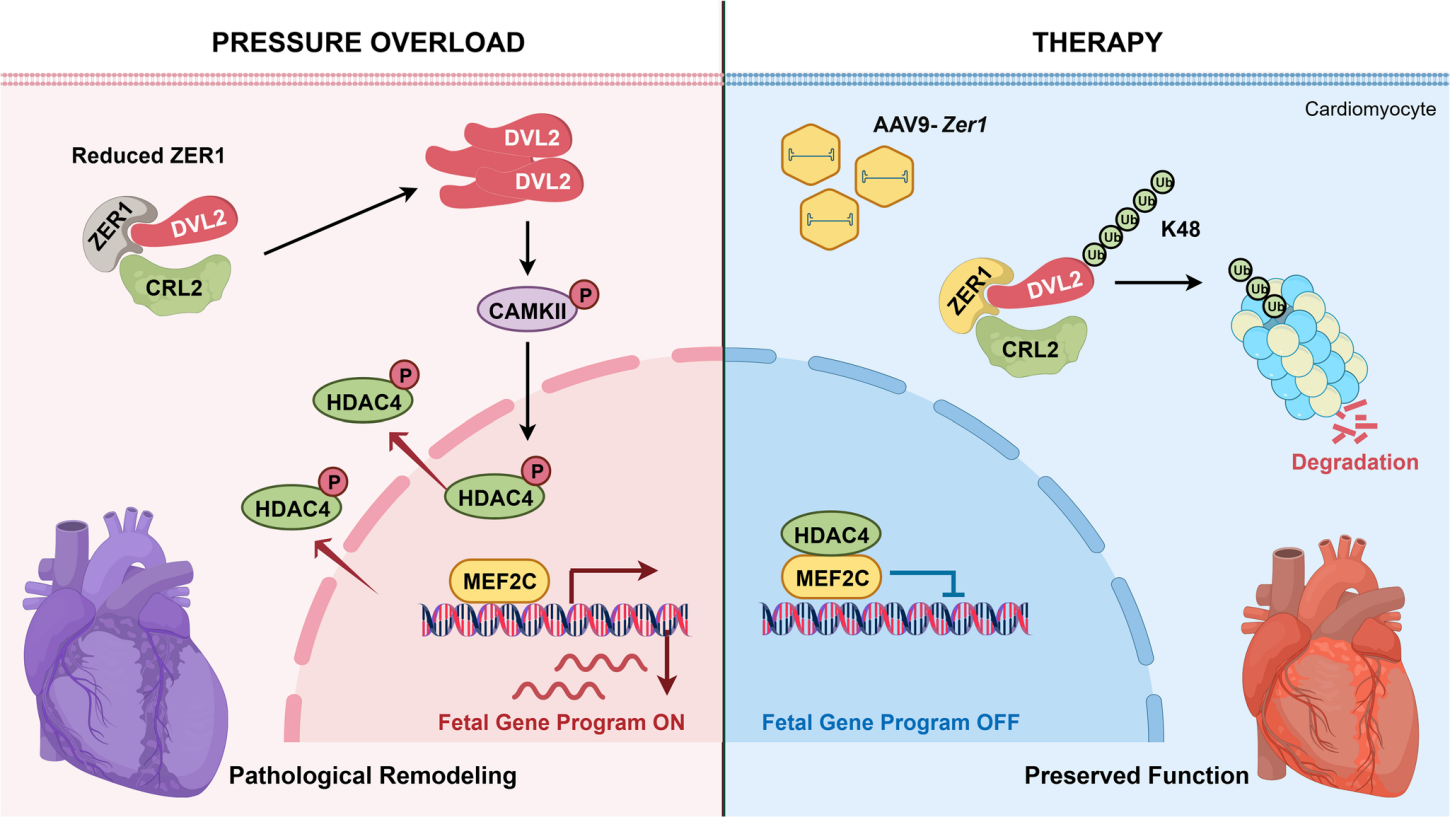
Working model of ZER1‐DVL2 regulation in pressure overload‐induced cardiac remodeling. Schematic model showing that pressure overload reduces cardiomyocyte ZER1, thereby weakening CRL2^ZER1^‐dependent DVL2 degradation and allowing DVL2 accumulation. Increased DVL2 activates the CaMKII‐HDAC4‐MEF2C axis and promotes fetal gene reactivation and pathological remodeling. Cardiomyocyte‐directed AAV9‐*Zer1* restoration enhances K48‐linked DVL2 polyubiquitination and proteasomal degradation, thereby restraining this signaling axis and preserving cardiac structure and function.

## 3 Discussion

In this study, we identify ZER1 as a previously unrecognized Gly/N‐degron proteostatic regulator that restrains pressure overload‐induced cardiac remodeling. ZER1 was selectively and consistently reduced within the CRL2/Gly‐N‐degron module in failing human hearts and hypertrophic mouse hearts. Mechanistically, ZER1 directly recognizes DVL2 in an N‐terminus‐dependent manner and promotes its K48‐linked proteasomal degradation, thereby limiting DVL2‐CaMKII‐HDAC4‐MEF2C signaling. Cardiomyocyte‐specific *Zer1* deletion exacerbated TAC‐induced remodeling, whereas cardiomyocyte‐targeted *Dvl2* knockdown eliminated the phenotypic differences between *Zer1* ^fl/fl^ and *Zer1*‐cKO mice, establishing DVL2 as a key downstream mediator of ZER1 loss. Finally, WWP1 knockdown failed to compensate for *Zer1* loss, whereas therapeutic cardiomyocyte‐selective ZER1 restoration attenuated established remodeling.

A central conceptual advance of this work is the implication of the Gly/N‐degron branch of the N‐degron system in adult cardiac remodeling. While other N‐degron branches have been linked to cardiovascular development and homeostasis through regulated proteolysis of defined substrates, the Gly/N‐degron pathway has remained largely unexplored in pressure overload‐induced remodeling [19, 20, 21, 22, 44, 45]. The novelty of this work lies not in re‐identifying DVL2‐MEF2C signaling as pro‐remodeling, but in defining a previously unrecognized Gly/N‐degron‐dependent degradation mechanism that restrains DVL2 dosage in the stressed adult heart. Our results support a model in which ZER1‐dependent substrate recognition provides a proteostatic checkpoint during mechanical stress, limiting accumulation of remodeling‐promoting signals. The observation that ZER1 is reduced in failing human myocardium and in cardiomyocytes from TAC hearts further suggests that diminished Gly/N‐degron capacity may accompany pathological remodeling and contribute to sustained signaling activation. ZYG11B is another Gly/N‐degron substrate receptor and may provide overlapping or substrate‐selective recognition within the same pathway. However, ZER1 was the only Gly/N‐degron receptor concordantly reduced across human DCM and mouse TAC datasets, supporting ZER1 as the disease‐associated receptor prioritized in this study. Whether ZYG11B compensates for ZER1 loss or controls distinct cardiac substrates remains an important question for future work. In parallel, endogenous cardiac ZER1 IP‐MS, N‐terminal degron compatibility, remodeling‐relevant prioritization, and targeted validation converged on DVL2 as a high‐priority ZER1 interactor. This strategy links cardiac‐context discovery with N‐degron biochemical logic and mitigates post hoc candidate selection.

DVL proteins integrate WNT and noncanonical signaling, and their abundance is tightly controlled by ubiquitin‐dependent turnover and other degradative routes [46, 47]. Multiple E3 ligases and deubiquitinating enzymes regulate DVL stability through distinct chain architectures, implying that ubiquitin linkage topology can encode divergent functional outcomes [36, 37, 48, 49, 50]. In cardiomyocytes, we previously showed that WWP1 stabilizes DVL2 via atypical K27‐linked ubiquitination and promotes remodeling after TAC [33]. In contrast, the present study identifies canonical K48‐linked ubiquitination as a dominant route for DVL2 turnover downstream of ZER1. These findings support a model in which DVL2 abundance in the stressed heart is governed by opposing dosage‐control mechanisms, with WWP1 providing a stabilizing input and ZER1 driving K48‐linked proteasomal degradation. In this context, the inability of *Wwp1* knockdown to rescue *Zer1*‐KO remodeling indicates that the protective effect of *Wwp1* depletion requires intact ZER1‐dependent DVL2 turnover during pressure overload‐induced cardiac remodeling.

Our interaction mapping also identifies a previously unrecognized determinant of DVL2 recognition. Consistent with prior evidence that ZER1‐family substrate receptors use ARM repeats to engage Gly/N‐degrons, we found that DVL2 binding depends on the local sequence context of its extreme N terminus: substitution of Ala1 and/or Gly2 with Asn was sufficient to abolish binding in cells and markedly weaken binding of synthetic DVL2 N‐terminal peptides in vitro [26]. Structural prediction supported accommodation of the DVL2 N terminus within an acidic pocket formed by the ZER1 ARM‐repeat region, and truncation mapping indicated that an intact ARM‐repeat region is required for DVL2 interaction in cells. This N‐terminal identity‐based recognition differs from mechanisms in which DVL regulation is mediated through internal motifs or domain‐centric interfaces, and it provides a distinct layer of substrate selectivity for DVL2 control [33, 48, 49, 51].

This study has limitations. First, although multiple orthogonal assays support N‐terminus‐dependent recognition, we did not directly interrogate upstream processing events or the enzymatic determinants that shape the relevant N‐terminal state in cardiomyocytes; accordingly, our conclusions focus on residue requirements for binding and degradation rather than regulation of N‐terminal processing per se. Second, ZER1 is a substrate receptor with the potential to regulate multiple targets; while the Dvl2 knockdown epistasis strongly supports a dominant role for DVL2 under TAC, additional ZER1‐dependent substrates may contribute to specific features of remodeling. Finally, although human myocardium analyses support disease association, larger cohorts with refined etiology stratification will be important to define the clinical generalizability of ZER1 downregulation.

In summary, our findings define ZER1 as a cardioprotective substrate receptor that limits pressure overload‐induced remodeling by promoting K48‐linked ubiquitination and proteasomal degradation of DVL2, thereby restraining CaMKII‐HDAC4 signaling and MEF2C‐driven transcriptional reprogramming. This work expands the scope of N‐degron biology in the cardiovascular system by implicating the Gly/N‐degron pathway in adult cardiac remodeling and positions ZER1‐dependent DVL2 degradation as a dominant proteostatic checkpoint with interventional relevance in pressure overload‐driven heart failure.

## 4 Methods

### 4.1 Human Heart Specimens

Left ventricular myocardial tissues were obtained from patients undergoing heart transplantation for heart failure (left ventricular ejection fraction < 40%) at Sun Yat‐Sen Memorial Hospital. Four non‐failing donor hearts from individuals who died in traffic accidents served as controls. Written informed consent was obtained from all participants or their legal representatives. The study complied with the Declaration of Helsinki and was approved by the Ethics Committee of Sun Yat‐Sen Memorial Hospital (Approval No: SYSKY‐2025‐304‐01).

### 4.2 Mice

Mice were housed in a specific pathogen‐free facility (23°C ± 3°C, 12 h light/dark cycle) with ad libitum access to food and water. All animal procedures were approved by the Experimental Animal Ethics Committee of Oujiang Laboratory (Approval No: OJLAB24020101).

#### 4.2.1 *Zer1* Global Knockout (KO)

*Zer1* KO mice on a C57BL/6J background were generated using CRISPR/Cas9 (GemPharmatech, Nanjing, Jiangsu). The targeted deletion spanned exons 3–9, resulting in loss of ZER1 protein. Founder genotypes were validated by sequencing. PCR genotyping was performed using primer sets: F1: 5’‐GTACATGGGCCAGCATTATGTGC‐3’ and R1: 5’‐GAAAGTCTTCTGAAAGCCAGGCG‐3’; F2: 5’‐TGCACTTTTGAGCCACACGAGAC‐3’ and R2: 5’‐TCGAAGGTGAAGTCCTTGACCAG‐3’. Unless stated otherwise, experiments were performed in 8‐week‐old male mice. For baseline aging experiments, WT and global *Zer1*‐KO mice were maintained under standard housing conditions and analyzed at 8 months of age without surgical intervention.

#### 4.2.2 Cardiomyocyte‐Specific *Zer1* Conditional KO (cKO)

*Zer1* ^fl/fl^ mice were crossed with Myh6‐Cre mice to generate *Zer1*‐cKO animals. Cardiac‐specific deletion was confirmed by RT‐qPCR and immunoblotting.

### 4.3 Transverse Aortic Constriction (TAC)

Pressure overload was induced by TAC as previously described with minor modifications [52]. Briefly, 8‐week‐old male mice (24–26 g) were anesthetized by intraperitoneal injection of 2,2,2‐tribromoethanol (0.1 mL/10 g). After thoracotomy, the aortic arch between the innominate and left common carotid arteries was constricted with a 6‐0 nylon suture tied against a blunted 27‐gauge needle; the needle was then removed. Sham controls underwent the same procedure without ligation.

### 4.4 Echocardiography

Transthoracic 2D guided M‐mode echocardiography was performed using a Vevo F2 system (FUJIFILM) with a 46 MHz transducer. Mice were anesthetized with isoflurane (2% induction, 1% maintenance) in 0.8 L/min oxygen on a warmed platform with ECG monitoring. LV dimensions and wall thickness were measured from at least three consecutive cardiac cycles and averaged. LV ejection fraction (EF) and fractional shortening (FS) were calculated from M‐mode parameters. Image acquisition and analysis were performed blinded to the experimental group whenever feasible.

### 4.5 Adult Cardiomyocyte and Non‐Cardiomyocyte Isolation

Adult cardiomyocytes were isolated using a Langendorff perfusion‐based enzymatic digestion method as previously described [53]. Following digestion and gentle trituration, cardiomyocytes were enriched by low‐speed sedimentation. The supernatant containing non‐cardiomyocytes was collected as the non‐cardiomyocyte fraction. Fractions were processed immediately for immunoblotting.

### 4.6 Histology and Morphometric Analyses

Hearts were imaged grossly using a stereomicroscope (Axio Zoom.V16, ZEISS), fixed, paraffin‐embedded, and sectioned at 5 µm. Sections were stained with hematoxylin and eosin (H&E), wheat germ agglutinin (WGA), and Masson's trichrome according to standard protocols. Fibrosis area and cardiomyocyte CSA were quantified in ImageJ using predefined thresholds. For CSA, five hearts per group were analyzed with ten randomly selected fields per heart.

### 4.7 Immunohistochemistry (IHC)

Sections underwent antigen retrieval in 0.01 M sodium citrate buffer (pH 6.0) by microwaving for 15 min. Endogenous peroxidase was quenched using 3% H_2_O_2_. Primary antibodies against ZER1 (Proteintech, Wuhan, Hubei, China; #16647‐1‐AP, 1:100) or DVL2 (Cell Signaling Technology, Boston, MA, USA; #3224S, 1:100) were incubated overnight at 4°C. Detection was performed with biotinylated secondary antibodies and DAB substrate (Zhongshan Golden Bridge, Beijing, China). Negative controls omitted primary antibody. For quantification, ZER1‐ or DVL2‐positive areas were measured in randomly selected fields using ImageJ and expressed as the percentage of the analyzed myocardial area.

### 4.8 Immunofluorescence (IF) and Immunocytochemistry

For tissue immunofluorescence, paraffin sections were deparaffinized, rehydrated, and subjected to antigen retrieval as described above, followed by permeabilization/blocking and incubation with primary antibodies. Cardiomyocytes were identified using cardiac troponin T (Thermo Fisher Scientific, Waltham, MA, USA; MA5‐12960, 1:100) where indicated, and nuclei were counterstained with DAPI. Fluorophore‐conjugated secondary antibodies were applied, and images were acquired by confocal microscopy.

For cell immunocytochemistry, cells were fixed in 4% paraformaldehyde (15 min), permeabilized/blocked in PBS containing 1% BSA and 0.1% Triton X‐100 (30 min), and incubated with primary antibodies overnight at 4°C. Antibodies included: ZER1 (Proteintech, #68258‐1‐Ig, 1:200), DVL2 (Cell Signaling Technology, #3224S, 1:200), HA (Abclonal, Wuhan, Hubei, China; #AE036, 1:200), Flag (Cell Signaling Technology, Boston, MA, USA; #8146S, 1:1000), and α‐actinin (Sigma Aldrich, Saint Louis, MO, USA; #A7811, 1:200). Secondary antibodies (ZSGB‐BIO, #ZF‐0316/#ZF‐0312) were used at 1:100. Images were acquired on a confocal microscope (FV3000, Olympus).

### 4.9 Flow Cytometry of hiPSC‐Derived Cardiomyocytes

hiPSC‐CMs were fixed (4% paraformaldehyde, 20 min), permeabilized (0.1% Triton X‐100, 15 min), and stained with anti‐cardiac troponin T (Miltenyi Biotec, #130‐119‐575, 1:100) in 0.1% BSA at 37°C for 60 min. Cells were analyzed by flow cytometry after resuspension in 0.5% BSA.

### 4.10 RNA Extraction and RT‐qPCR

Total RNA was extracted using TRIzol (Takara, Kyoto, Japan). cDNA was synthesized from 1 µg RNA using HiScript III qRT SuperMix (+gDNA wiper; Vazyme, Nanjing, Jiangsu). qPCR was performed using SYBR Green chemistry (Vazyme) on a Roche LightCycler system. Relative expression was calculated by the 2^−ΔΔCt^ method normalized to *Gapdh* (mouse) or *GAPDH* (human).

**Table:** 

Mouse *Zer1*	forward primer	5’‐GGAACTGCTGTCTGACCCTC‐3’
	reverse primer	5’‐TCGCAGTTGTCAGGTGTCTC‐3’
Mouse *Dvl2*	forward primer	5’‐GCTGCCCACCTTCTCCTAC‐3’
	reverse primer	5’‐CCCATCGCTTCTTGTCGAC ‐3’
Mouse *Nppa*	forward primer	5’‐TTCGGGGGTAGGATTGACAG‐3’,
	reverse primer	5’ ‐CACACCACAAGGGCTTAGGA‐3’
Mouse *Nppb*	forward primer	5’ ‐TGTTTCTGCTTTTCCTTTATCTG‐3’
	reverse primer	5’‐TCTTTTTGGGTGTTCTTTTGTGA‐3’
Mouse *Myh7*	forward primer	5'‐CCTCAGCAGAGGAGTACAGC‐3’
	reverse primer	5'‐GGCTGAGCCTTGGATTCTCA‐3’
Mouse *Axin2*	forward primer	5’‐CGCCTAGTGACTGCTGGAAA‐3’
	reverse primer	5’‐ACGGAAAACAACGATCCCGA‐3’
Mouse *Nkd1*	forward primer	5’‐CTCAGGGCTTGGGGAAAAGA‐3’
	reverse primer	5’‐GACGCTCCTCTTACTCTGGC‐3’
Mouse *Ccnd1*	forward primer	5’‐AAAATGCCAGAGGCGGATGA‐3’
	reverse primer	5’‐GAGGGGGTCCTTGTTTAGCC‐3’
Mouse *Wwp1*	forward primer	5’‐GAGGGGCAGAGCCGTTAAT‐3’
	reverse primer	5’‐TCAGCTGCTCGTCCCATTTT‐3’
Mouse *Gapdh*	forward primer	5’‐ACTCCACTCACGGCAAATTCA ‐3’
	reverse primer	5’‐GGCCTCACCCCATTTGATG‐3’
Human *DVL2*	forward primer	5’‐CCCCATTGTGCTGACTGTG‐3’
	reverse primer	5’‐GCAAAGACGATCCAGATGTAAT‐3’
Human *ZER1*	forward primer	5’‐GGGCTGTGAAGATGAGTACCTC‐3’
	reverse primer	5’‐GCTGTCTTTCCACTGGGTGA‐3’
Human *NPPA*	forward primer	5’‐CTAAAAAGCAAGCTGAGGGCG‐3’
	reverse primer	5’‐ACAGGAGCCTCTTGCAGTCT‐3’
Human *NPPB*	forward primer	5’‐TGGAAACGTCCGGGTTACAG‐3’
	reverse primer	5’‐CTTCCAGACACCTGTGGGAC‐3’
Human *MYH7*	forward primer	5’‐GAGGAGCAAGCCAACACCAA‐3’
	reverse primer	5’‐ACTCCTCATTCAAGCCCTTCG‐3’
Human *GAPDH*	forward primer	5’‐GTCTTCACCACCATGGAGAAGG‐3’
	reverse primer	5’‐GCCTGCTTCACCACCTTCTTGA‐3’

### 4.11 Public Transcriptome Analysis

Analyses were performed in R using public GEO datasets.

#### 4.11.1 Human Cohort

For human left ventricular RNA‐seq data (GSE116250), normalized expression values supplied by the dataset (RPKM) were log_2_‐transformed after adding a pseudocount of 1. Group comparisons (DCM vs. non‐failing) were performed using two‐sided Mann‐Whitney U tests. A CRL2/Gly‐N‐degron module score was computed per sample by z‐scoring expression of *ZER1*, *CUL2*, *ELOC*, *ELOB*, *RBX1*, and *ZYG11B* within the dataset and averaging the z‐scores. A fetal gene program score was calculated as the mean z‐score of *NPPA*, *NPPB*, and *MYH7*. Associations between module and fetal gene scores were assessed using Pearson correlation.

#### 4.11.2 Mouse Cohort

For mouse pressure‐overload RNA‐seq data (GSE203083), log_2_(FPKM) values were used. The module score was computed analogously using *Zer1*, *Cul2*, *Eloc*, *Elob*, *Rbx1* and *Zyg11b*. Group comparisons (TAC vs sham) used two‐sided Mann–Whitney U tests, and correlations used Pearson correlation.

### 4.12 ZER1 Immunoprecipitation‐Mass Spectrometry (IP‐MS)

Mouse left ventricular lysates were subjected to immunoprecipitation using anti‐ZER1 (Proteintech, #16647‐1‐AP) or control IgG (Abclonal, #AC005). Immunoprecipitates were processed by FASP (10 kDa cutoff filters; Sartorius, Gottingen, Niedersachsen, Germany), reduced with DTT, alkylated with acrylamide, and digested with trypsin overnight at 37°C. Peptides were separated on a Vanquish NEO nanoLC system coupled to an Orbitrap Exploris 480 (Thermo Fisher Scientific) operated in data‐dependent acquisition. Raw files were searched in Proteome Discoverer against the UniProt human database with a 1% FDR. Carbamidomethylation was set as fixed modification; oxidation (M), phosphorylation (S/T/Y), protein N‐terminal acetylation, and pyroglutamate conversion were considered variable modifications.

### 4.13 Predefined Candidate Prioritization and Tiering for ZER1 IP‐MS Interactors

To minimize post hoc selection, candidates enriched in ZER1‐IP vs. IgG were subjected to a predefined prioritization. N‐terminus compatibility was assessed in silico using established initiator methionine excision rules to annotate candidates with putative exposed N‐terminal residues compatible with Gly/N‐degron recognition [23]. N‐terminus‐compatible candidates were then tiered using predefined Gene Ontology Biological Process keyword sets related to cardiac remodeling, yielding Tier 1 and Tier 2 candidate groups for prioritization and visualization. This tiering strategy was used to guide targeted validation and was not used to define statistical significance.

### 4.14 Cell Culture and Transfection

HEK293T cells were cultured in DMEM (Gibco, Grand Island, NY, USA) with 10% FBS and 1% penicillin/streptomycin. HL‐1 cardiomyocytes were cultured in Claycomb medium (Sigma) supplemented with norepinephrine, FBS, penicillin/streptomycin, and L‐glutamine.

Neonatal mouse cardiomyocytes were isolated from 1 to 3‐day‐old pups by enzymatic digestion and cultured in DMEM with 10% FBS. For in vitro hypertrophy assays, NMCMs isolated from *Zer1* ^fl/fl^ and *Zer1*‐cKO pups were treated with PBS or angiotensin II (AngII; 1 µm, 48 h).

Plasmid transfections used Lipofectamine 3000 (Thermo Fisher). siRNA transfections used Lipofectamine RNAiMAX. hiPSC‐CMs (HELP Therapeutics; internal number XYJ010001/L18C) were cultured under standard conditions (37°C, 5% CO_2_) with medium changes every 48 h.

### 4.15 Plasmids and siRNAs

Mouse and human ZER1 expression plasmids were obtained from a plasmid library (PPL, China). ZER1 truncations (F1‐F4), GST‐ZER1, and DVL2 point mutants (A1N, G2N, A1N/G2N) were generated by site‐directed mutagenesis. All other plasmids used in this study have been described in reference [33]. siRNAs were synthesized by GenePharma with sequences as follows:

Negative control:
5’‐UUCUCCGAACGUGUCACGUTT‐3’
Human *ZER1* siRNA:
5’‐CUCGGAGAAACAUCAAUUATT‐3’
Human *DVL2* siRNA:
5’‐UCCACAAUGUCUCUCAAUA‐3’
Mouse *Zer1* siRNA:
5’‐GUGAUGCCACUUUCCUAACTT‐3’
Mouse *Dvl2* siRNA:
5’‐TCCACAATGTCTCTCAACA‐3’

### 4.16 AAV9 Vectors and In Vivo Delivery

AAV9 vectors were generated by OBiO Technology. For cardiomyocyte‐specific *Zer1* overexpression, mouse *Zer1* cDNA was cloned downstream of the cTnT promoter, with a matching empty vector used as control. For cardiomyocyte‐specific *Dvl2* knockdown, a miR30‐based shRNA cassette targeting mouse *Dvl2* was expressed under the cTnT promoter, with a non‐targeting shRNA used as control. For *Wwp1* knockdown, an shRNA cassette targeting mouse *Wwp1* was expressed from the cTnT promoter, and EGFP was included as a reporter. Mice received 5.0 × 10^11^ vector genomes per mouse via tail vein injection. For post‐TAC intervention protocols, AAV9 vectors were administered 1 week after TAC or sham surgery, and mice were analyzed 3 weeks later unless otherwise stated.

### 4.17 Cycloheximide Chase

HEK293T cells were transfected with Flag‐DVL2 ± HA‐ZER1 (24 h) or siRNA targeting *ZER1* (48 h), then treated with cycloheximide (CHX, MedChemExpress, NJ, USA; 50 µm) for the indicated durations. For cardiomyocyte CHX chase assays, NMCMs were isolated from WT and global *Zer1*‐KO mice and treated with CHX (50 µm) for the indicated durations. Lysates were collected at each time point and analyzed by immunoblotting; DVL2 intensities were normalized to GAPDH.

### 4.18 Pharmacological and Amino‐Acid Treatments

Where indicated, cells were treated with MG132 (10 µm, 6 h), MLN4924 (10 µm, 24 h), or phenylalanine (Phe, Sigma Aldrich; 20 µm, 24 h). For free amino‐acid supplementation assays, glycine or alanine (10 mm) was added for 8 h prior to harvest.

### 4.19 Immunoblotting

For mouse myocardial samples, left ventricular tissues were collected, rapidly rinsed in cold PBS, snap‐frozen, and used for protein extraction unless otherwise specified. Tissues and cells were lysed in RIPA buffer (Solarbio, Beijing, China) supplemented with protease/phosphatase inhibitors (Roche, Basel, Switzerland). Lysates were cleared by centrifugation, quantified by BCA assay, separated by SDS‐PAGE, and transferred to PVDF membranes. Membranes were blocked (5% milk, 1 h), incubated with primary antibodies overnight at 4°C, then with HRP‐conjugated secondary antibodies. Signals were detected by ECL and quantified in ImageJ.

Antibodies used were: anti‐GAPDH (Abways Technology, Shanghai, China; #AB0037, 1:5000), anti‐ZER1 (Proteintech, #16647‐1‐AP, 1:1000), anti‐DVL2 (Cell Signaling Technology, #3224S, 1:1000), anti‐HADC4 (4A3)(Cell Signaling Technology, #5392, 1:1000), anti‐Phospho‐HDAC4(Ser246)(Cell Signaling Technology, #3443, 1:1000), anti‐CaMK2 alpha/delta (GeneTex, San Antonio, TX, USA; #GTX52377, 1:1000), anti‐CaMK2 beta/gamma/delta (phosphor Thr287, GeneTex, GTX52342, 1:1000), anti‐Ubiquitin Polyclonal (Proteintech, #10201‐2‐AP, 1:1000), anti‐WWP1 (ABclonal, #A5269, 1:1000), anti‐active β‐catenin (Cell Signaling Technology, #8814, 1:1000), anti‐total β‐catenin (Proteintech, 66379‐1‐Ig, 1:5000), anti‐EZR (Proteintech, 26056‐1‐AP, 1:20000), anti‐DVL1 (Proteintech, 27384‐1‐AP, 1:1000), anti‐DVL3 (Proteintech, 13444‐1‐AP, 1:2000). anti‐DYKDDDDK Tag (9A3) (Cell Signaling Technology, #8146, 1:1000), anti‐HA‐Tag (MBL, Beijing, China; #M180‐3, 1:1000), anti‐His‐Tag (Proteintech, #66005‐1‐Ig, 1:5000), anti‐GST‐Tag (Cell Signaling Technology, #2622, 1:1000), HRP‐labeled Goat Anti‐Mouse IgG(H+L)(Beyotime, #A0216, 1:5000), HRP‐labeled Goat Anti‐Rabbit IgG(H+L))(Beyotime, Shanghai, China; #A0208, 1:10000), Anti‐mouse IgG for IP (HRP)(Abcam, Cambridge, UK; #ab131368, 1:5000).

### 4.20 Immunoprecipitation (Co‐IP)

Cells were lysed in NP‐40 IP buffer (20 mm Tris‐HCl pH 7.4, 1% NP‐40, 10% glycerol, 135 mm NaCl) with protease/phosphatase inhibitors. Lysates were incubated with primary antibody overnight at 4°C, followed by Protein A/G agarose beads (2 h). Beads were washed four times, eluted in SDS sample buffer, and analyzed by immunoblotting.

### 4.21 In Vivo Ubiquitination Assay

HEK293T cells were transfected with HA‐ZER1, Flag‐DVL2, and HA‐ubiquitin constructs. After 24 h, cells were lysed in ubiquitination lysis buffer (50 mm Tris‐HCl pH 7.4, 150 mm NaCl, 1% Triton X‐100, 5 mm EDTA, 0.1% SDS, 0.5% sodium deoxycholate) with inhibitors. Flag‐DVL2 was immunoprecipitated (anti‐Flag; CST #8146S) and ubiquitinated species were detected by anti‐ubiquitin immunoblotting.

### 4.22 Protein Purification and GST Pull‐Down

GST‐ZER1 and His‐DVL2 were expressed in E. coli and purified using glutathione‐Sepharose and Ni‐NTA beads, respectively, following established protocols. For pull‐down, GST or GST‐ZER1 immobilized on glutathione beads was incubated with purified His‐DVL2 or with lysates from Flag‐DVL2‐transfected HEK293T cells. After washing, bound proteins were eluted and analyzed by immunoblotting.

### 4.23 Structural Prediction

Protein‐peptide interaction predictions were performed using AlphaFold3 (Google Colab implementation). ZER1 (full‐length) and a short DVL2 N‐terminal peptide sequence were provided as inputs. Structural visualization and interface inspection were performed in PyMOL.

### 4.24 Surface Plasmon Resonance (SPR)

SPR measurements were performed at 25°C on a Biacore 8K system (Cytiva, Medford, MA, USA) using CM5 sensor chips. Purified GST‐ZER1, His‐DVL2, and DVL2 N‐terminal degron peptides (WT, A1N, G2N, and A1N/G2N) were buffer‐exchanged into running buffer by ultrafiltration (Millipore, Bedford, MA, USA). Running buffer was phosphate‐buffered saline (PBS, pH 7.4; 10 mm phosphate, 137 mm NaCl, 2.7 mm KCl) supplemented with 0.05% Tween‐20. Proteins were immobilized on CM5 chips using standard amine‐coupling chemistry. Analytes were injected at serially diluted concentrations, and binding responses were recorded as sensorgrams. Two orientations were tested for protein‐protein binding: (i) immobilized GST‐ZER1 with His‐DVL2 as analyte, and (ii) immobilized His‐DVL2 with GST‐ZER1 as analyte. For peptide‐binding assays, GST‐ZER1 was immobilized and degron peptides were injected as analytes. Sensorgrams were reference‐subtracted and analyzed using Biacore 8K Insight Evaluation Software to obtain kinetic parameters and dissociation constants (K_D_) by fitting to a 1:1 Langmuir binding model.

### 4.25 Luciferase Reporter Assays

HL‐1 cells were transfected with p3×MEF2‐luc and pRL‐TK (Renilla control) with the indicated expression plasmids and/or siRNAs. After 48 h, luciferase activity was measured using the Dual‐Luciferase Reporter Assay System (Promega). Firefly luciferase was normalized to Renilla luciferase.

### 4.26 Statistics

Data are presented as mean ± SEM unless stated otherwise. Normality was assessed by Shapiro–Wilk test or Q‐Q plots. Two‐group comparisons used unpaired two‐tailed Student's *t*‐tests (normal distributions) or Mann–Whitney U tests (non‐normal distributions). Multi‐group comparisons used one‐way or two‐way ANOVA with Holm–Šídák multiple‐comparison correction. Homogeneity of variance was assessed prior to ANOVA. When variances were unequal, Welch's ANOVA was used for one‐way analyses and standard two‐way ANOVA was performed after log transformation. Pearson correlation was used for association analyses. All tests were two‐sided; *p* < 0.05 was considered significant. Analyses were performed in GraphPad Prism (v10.0) and/or R, as applicable.

## Author Contributions

S.L. conceived and supervised the study. Y.X.L., W.S., and Y.W. provided overall guidance and served as corresponding authors. M.J. designed the experiments and drafted the manuscript. M.J., Z.L., L.C., and Y.N. performed the majority of experiments and analyzed the data. J.Z. contributed to bioinformatic analyses and data interpretation. W.Z., H.W., P.Y., X.L., K.W., and R.Q. assisted with animal experiments and data acquisition. N.X. and X.M. contributed to molecular biology experiments. D.Z., J.L., G.Z., S.H., X.C., S.L., Y.L., Y.W., and Q.K. provided critical reagents, technical support, and methodological expertise. J.Z. and Y.F. collected human heart samples. All authors reviewed and approved the final version of the manuscript.

## Funding

This work was supported by the National Natural Science Foundation of China (grant 82471913 to Shukuan Ling and grant 82192882 to Yingxian Li), Zhejiang Provincial Natural Science Foundation of China (grant LZ23H210001 to Shukuan Ling), National Key Research and Development Project (grant 2022YFA1104203 to Yingxian Li), and the Summit Advancement Disciplines of Zhejiang Province (Wenzhou Medical University‐Pharmaceutics).

## Ethics Statement

Human studies: Ethics Committee of Sun Yat‐sen Memorial Hospital (SYSKY‐2025‐304‐01). Animal studies: Experimental Animal Ethics Committee of Oujiang Laboratory (OJLAB24020101).

## Conflicts of Interest

The authors declare no conflicts of interest.

## Supporting information




**Supporting File**: advs76308‐sup‐0001‐SuppMat.pdf.

## Data Availability

Publicly available transcriptome datasets analyzed in this study are deposited in NCBI Gene Expression Omnibus (GEO) under accession codes GSE116250 and GSE203083. The analysis code used for CRL2/Gly‐N‐degron module scoring, transcriptome analyses/plotting, and N‐terminus‐based candidate triage is available on Zenodo (https://doi.org/10.5281/zenodo.20487110). Proteomics‐derived ZER1 interactors and candidate prioritization outputs are provided in the Supporting Tables.
