# Supplementary material for: ZER1 Restrains Pressure Overload‐Induced Cardiac Remodeling by Targeting DVL2 for Gly/N‐Degron‐Dependent Degradation
Source: Adv Sci (Weinh). 2026 Jun 29:e76308. Online ahead of print. doi: 10.1002/advs.76308 (PMC13336824; doi:10.1002/advs.76308)
Supplement: Supplementary file 1 — Supporting File: advs76308‐sup‐0001‐SuppMat.pdf. [file ADVS-9999-e76308-s001.pdf]

SUPPLEMENTAL MATERIAL

ZER1 Restrains Pressure Overload-induced Cardiac Remodeling by Targeting DVL2 for Gly/N-degron-dependent Degradation

Mingchao Jiang[1,2#], Zhehao Lin[2#], Lu Chen[1,2#], Ying Ni[1#], Jun Zhou[1], Wenjuan Zhang[3], Huilin Wang[1], Peifeng Ying[1], Xiu Lu[1], Kai Wang[1], Qingran Kong[4,5], Naxin Xu[6], Dingsheng Zhao[7], Jianwei Li[7], Guohui Zhong[7], Xingchen Meng[1], Sarkawt Hamad[8], Junmeng Zheng[9], Yuan Fu[9], Rongjiang Qin[1], Xuran Chu[1], Shangxuan Li[1,2], Youyou Li[10], Yili Wu[1,2], Yi Wang[11*], Weihong Song[1,2*], Yingxian Li[7*], Shukuan Ling [1,2*]

Supplemental Figure 1

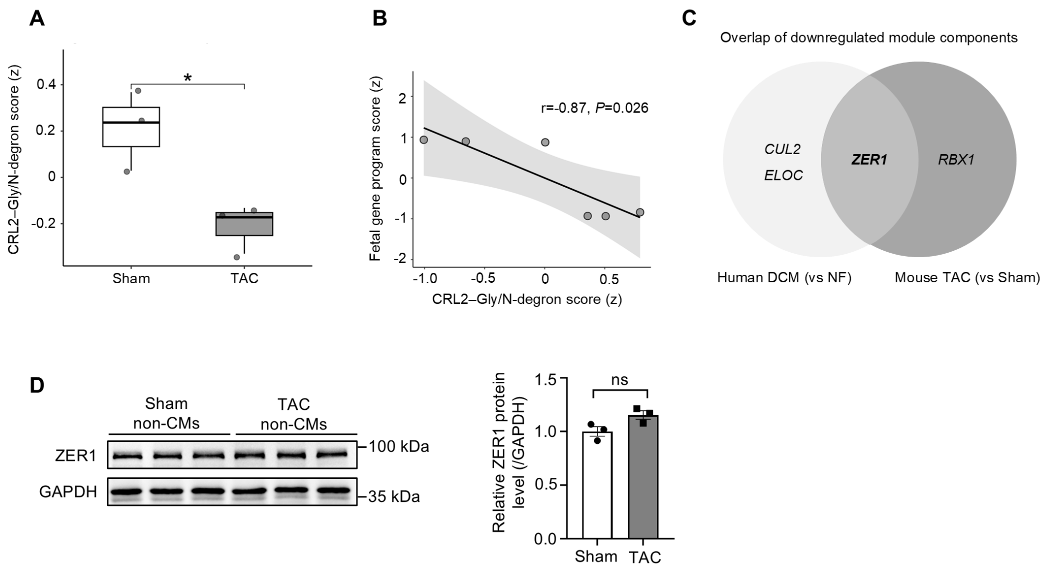

Supplementary Fig. 1. Conservation of CRL2/Gly-N-degron module alterations in mouse pressure overload. (A) CRL2/Gly-N-degron module score in mouse hearts subjected to sham or TAC in GSE203083. **(B)** Correlation between CRL2/Gly-N-degron module score and fetal gene program score based on *Nppa*, *Nppb*, and *Myh7* expression in mouse hearts from GSE203083. Pearson correlation with two-sided P value. **(C)** Venn diagram showing overlap of CRL2/Gly-N-degron module components significantly downregulated in human DCM hearts and mouse TAC hearts. **(D)** Immunoblot analysis and quantification of ZER1 in non-cardiomyocytes isolated from adult mice 4 weeks after sham or TAC (n=3 per group). Data are presented as mean ± SEM. Statistical analyses were performed using unpaired two-tailed Student's t tests or Mann-Whitney U test for two-group comparisons. ns, not significant; $^*P < 0.05$.

Supplemental Figure 2

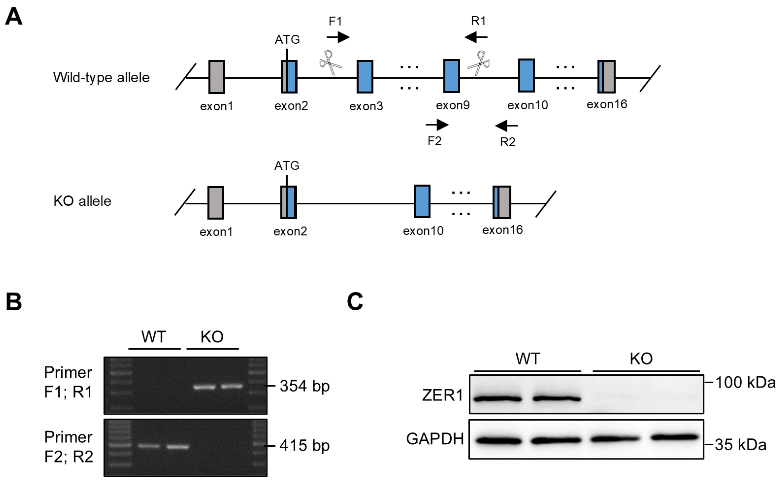

Supplementary Fig. 2. Validation and echocardiographic assessment of global *Zer1* knockout mice. (A) Strategy for generating global *Zer1* knockout (KO) mice. **(B)** PCR genotyping of WT and *Zer1* KO mice. **(C)** Immunoblot analysis of ZER1 in cardiac extracts from WT and *Zer1* KO mice.

Supplemental Figure 3

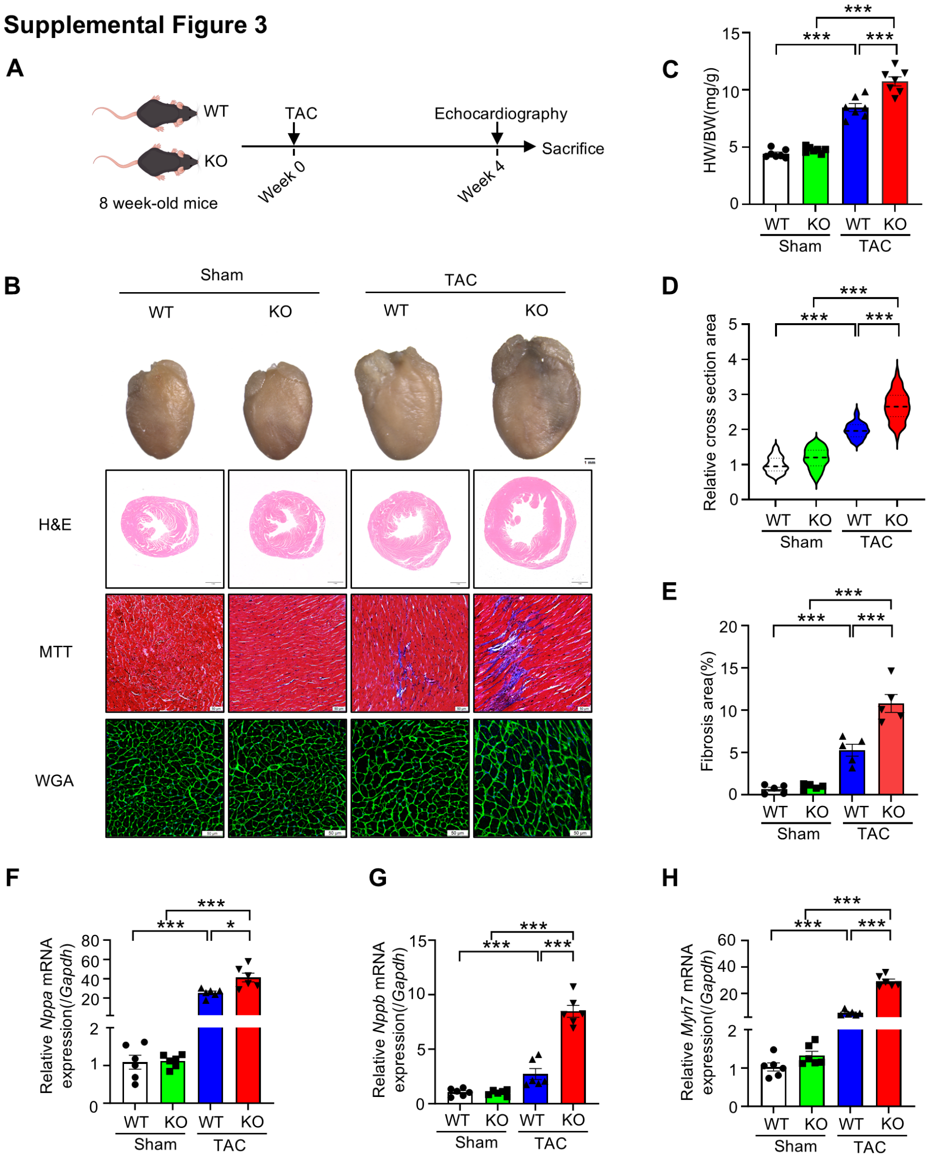

Supplementary Fig. 3. Global *Zer1* knockout aggravates pressure overload-induced cardiac remodeling. (A) Experimental timeline for sham or TAC surgery in WT and *Zer1* KO mice. **(B)** Representative gross hearts and histological analyses 4 weeks after sham or TAC. H&E, Masson's trichrome staining (MTT), and WGA staining are shown. Scale bars, 1 mm for H&E and 50 μm for MTT and WGA. **(C)** HW/BW ratios 4 weeks after sham or TAC (n=7 mice per group). **(D)** Quantification of cardiomyocyte cross-sectional area by WGA staining (n=5 hearts per group; 10 fields

per heart). **(E)** Quantification of myocardial fibrosis from MTT staining (n = 5 hearts per group). **(F-H)** RT-qPCR analysis of *Nppa*, *Nppb*, and *Myh7* mRNA expression in hearts 4 weeks after sham or TAC; values were normalized to *Gapdh* (n=6 mice per group). Data are presented as mean ± SEM. Statistical analyses were performed using two-way ANOVA followed by Holm-Šídák's post hoc test. *$P < 0.05$ and ***$P < 0.001$.

Supplemental Figure 4

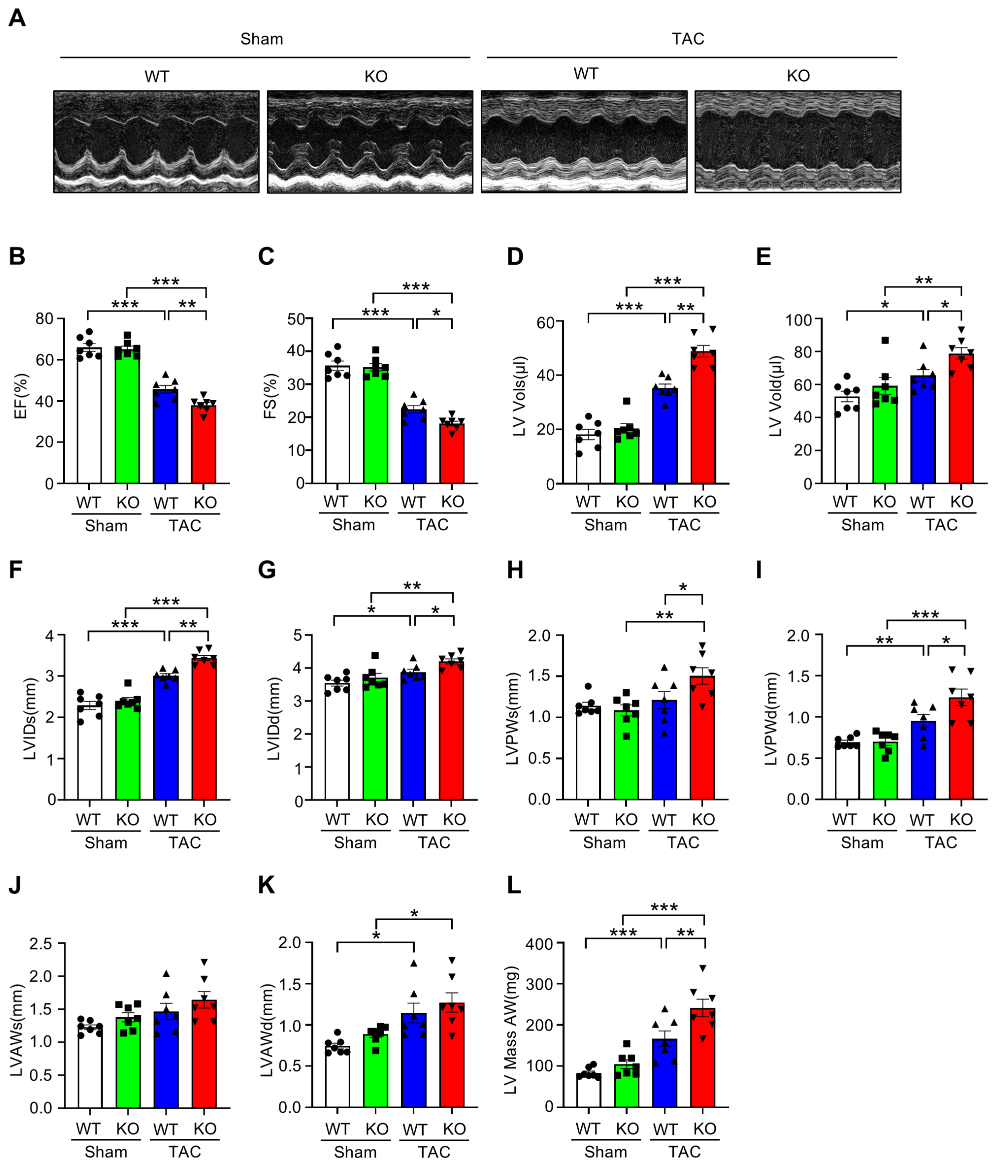

Supplementary Fig. 4. Global *Zer1* knockout aggravates pressure overload-induced cardiac dysfunction. (A) Representative M-mode echocardiographic images of WT and *Zer1* KO mice 4 weeks after sham or TAC. **(B-L)** Echocardiographic parameters in WT and *Zer1* KO mice 4 weeks after sham or TAC. Data are presented as mean ± SEM. Statistical analyses were performed using two-way ANOVA followed by Holm-Šídák's post hoc test. *$P < 0.05$, **$P < 0.01$, and ***$P < 0.001$.

Supplemental Figure 5

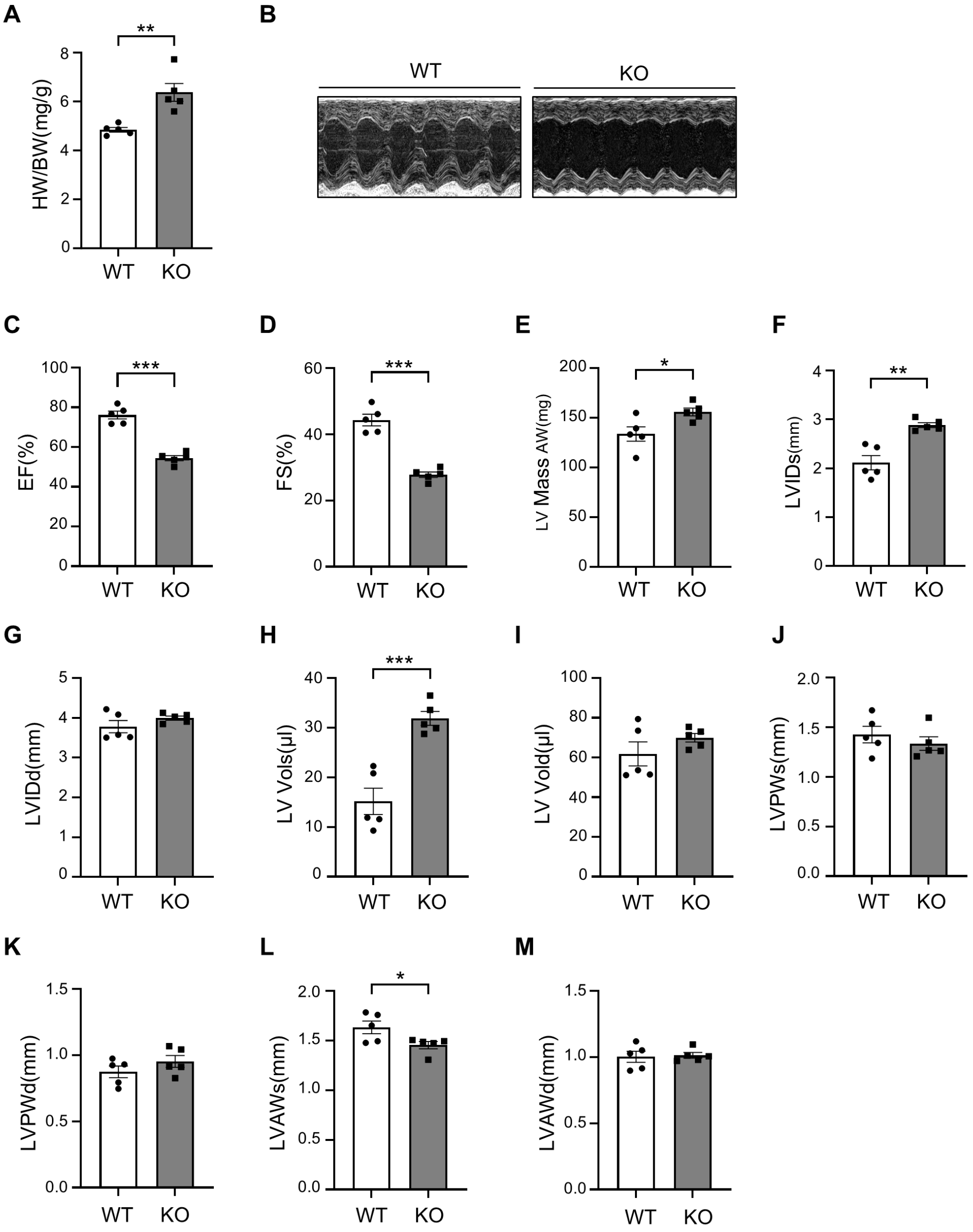

Supplementary Fig. 5. Global Zer1 knockout mice develop spontaneous cardiac remodeling with aging. (A) HW/BW ratio in 8-month-old WT and *Zer1* KO mice under basal conditions. **(B)** Representative M-mode echocardiographic images of 8-month-old WT and *Zer1* KO mice. **(C-M)** Echocardiographic assessment of cardiac function and remodeling in 8-month-old WT and *Zer1* KO mice under basal conditions.

Data are presented as mean ± SEM. Statistical analyses were performed using unpaired two-tailed Student's t tests. ns, not significant; *$P < 0.05$, **$P < 0.01$, and ***$P < 0.001$.

Supplemental Figure 6

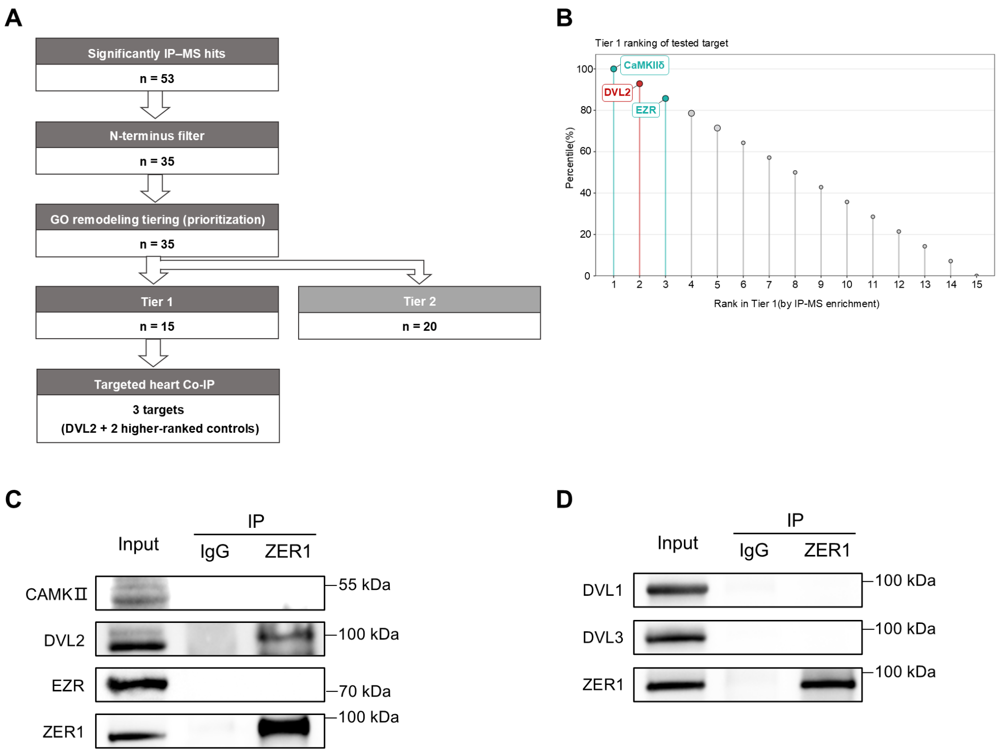

Supplementary Fig. 6. Cardiac IP-MS-guided prioritization and targeted validation of ZER1-associated candidates.

(A) Predefined prioritization workflow for endogenous cardiac ZER1 IP-MS candidates. Proteins significantly enriched in ZER1-IP relative to IgG-IP were filtered by N-terminus compatibility and then prioritized by GO remodeling-related annotation into Tier 1 and Tier 2 candidates. **(B)** Tier 1 ranking of tested candidates based on cardiac IP-MS enrichment. DVL2 and the higher-ranked Tier 1 candidates selected for targeted validation are highlighted. **(C)** Targeted endogenous Co-IP analysis of selected Tier 1 candidates in mouse heart lysates using anti-ZER1 or control IgG immunoprecipitation. **(D)** Endogenous Co-IP analysis of DVL family members in mouse heart lysates using anti-ZER1 or control IgG immunoprecipitation.

Supplemental Figure 7

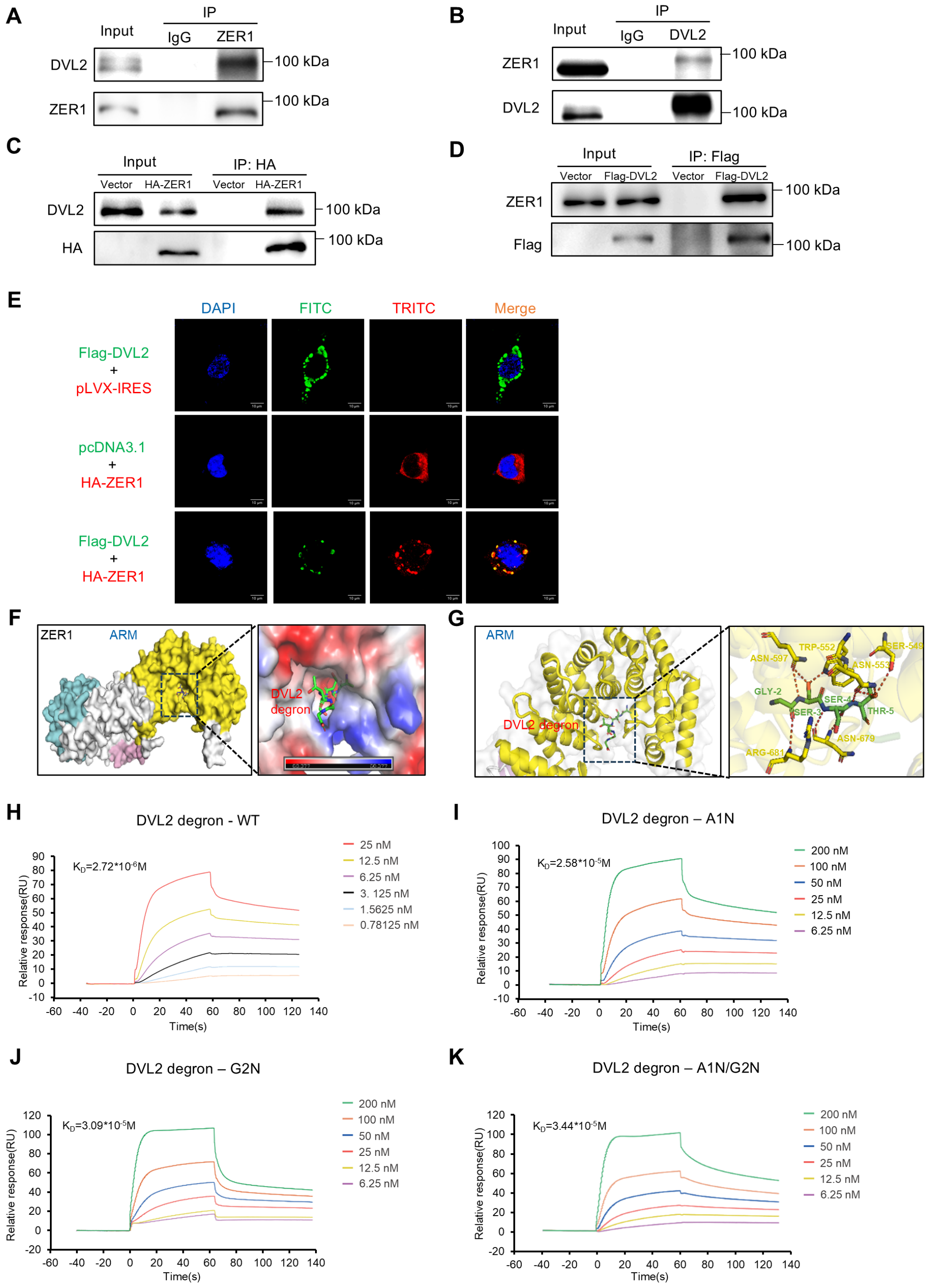

Supplementary Fig. 7. DVL2 directly interacts with ZER1 through its N-terminal degron.

(A, B) Endogenous Co-IP of ZER1 (A) and DVL2 (B) from HEK293T cells. **(C, D)** Semi-endogenous Co-IP assays in HEK293T cells following HA-ZER1 overexpression

(C) or Flag-DVL2 overexpression (D). **(E)** Confocal microscopy showing co-localization of ZER1 and DVL2 in HEK293T cells. Scale bar, 10 μm. **(F, G)** AlphaFold 3 structural prediction of ZER1 engagement with the DVL2 N-terminal degron. **(H-K)** SPR analysis of ZER1 binding to WT and mutant DVL2 N-terminal peptides. Dissociation constants (K_D) are indicated.

Supplemental Figure 8

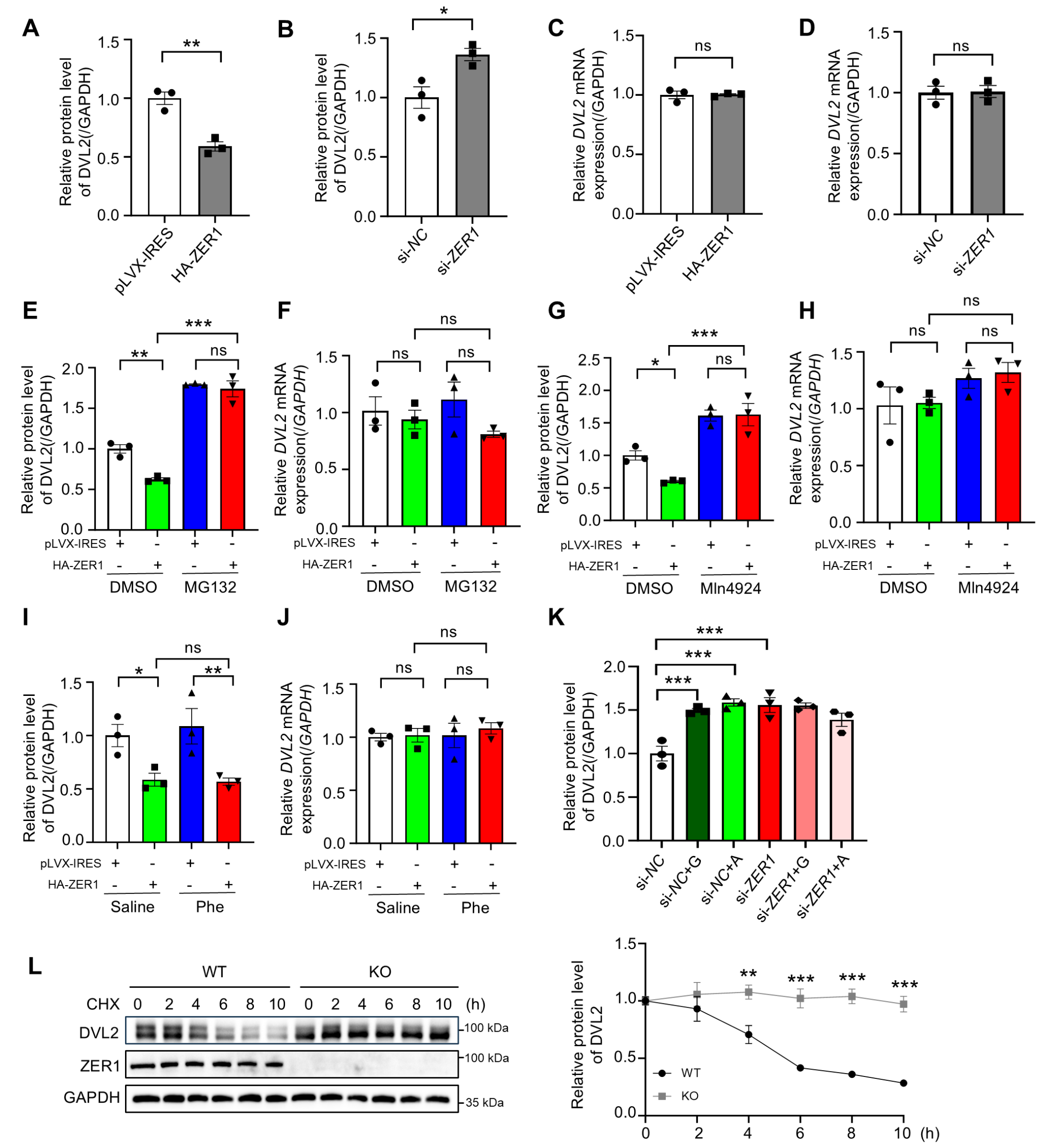

Supplementary Fig. 8. Additional quantification supporting ZER1-dependent DVL2 turnover.

(A, B) Quantification of DVL2 protein levels in HEK293T cells following HA-ZER1 overexpression (A) or ZER1 knockdown (B). **(C, D)** RT-qPCR analysis of *DVL2* mRNA levels following HA-ZER1 overexpression (C) or ZER1 knockdown (D). **(E, G, I)** Quantification of DVL2 protein levels in HEK293T cells overexpressing HA-ZER1 and treated with MG132 (10 μM) (E), MLN4924 (10 μM) (G), or Phe (20 μM)

(I). **(F, H, J)** RT-qPCR analysis of *DVL2* mRNA levels under MG132 (10 μM) (F), MLN4924 (10 μM) (H), or Phe (20 μM) (J) treatment. **(K)** Quantification of DVL2 protein levels following glycine or alanine supplementation in HEK293T cells with ZER1 knockdown or control siRNA. **(L)** CHX chase analysis and quantification of DVL2 turnover in neonatal mouse cardiomyocytes isolated from WT and *Zer1*-KO mice. Data are presented as mean ± SEM. Statistical analyses were performed using unpaired two-tailed Student's t tests or Mann-Whitney U test for two-group comparisons and two-way ANOVA followed by Holm-Šídák's post hoc test for multi-group comparisons. ns, not significant; $*P < 0.05$, $**P < 0.01$, and $***P < 0.001$.

Supplemental Figure 9

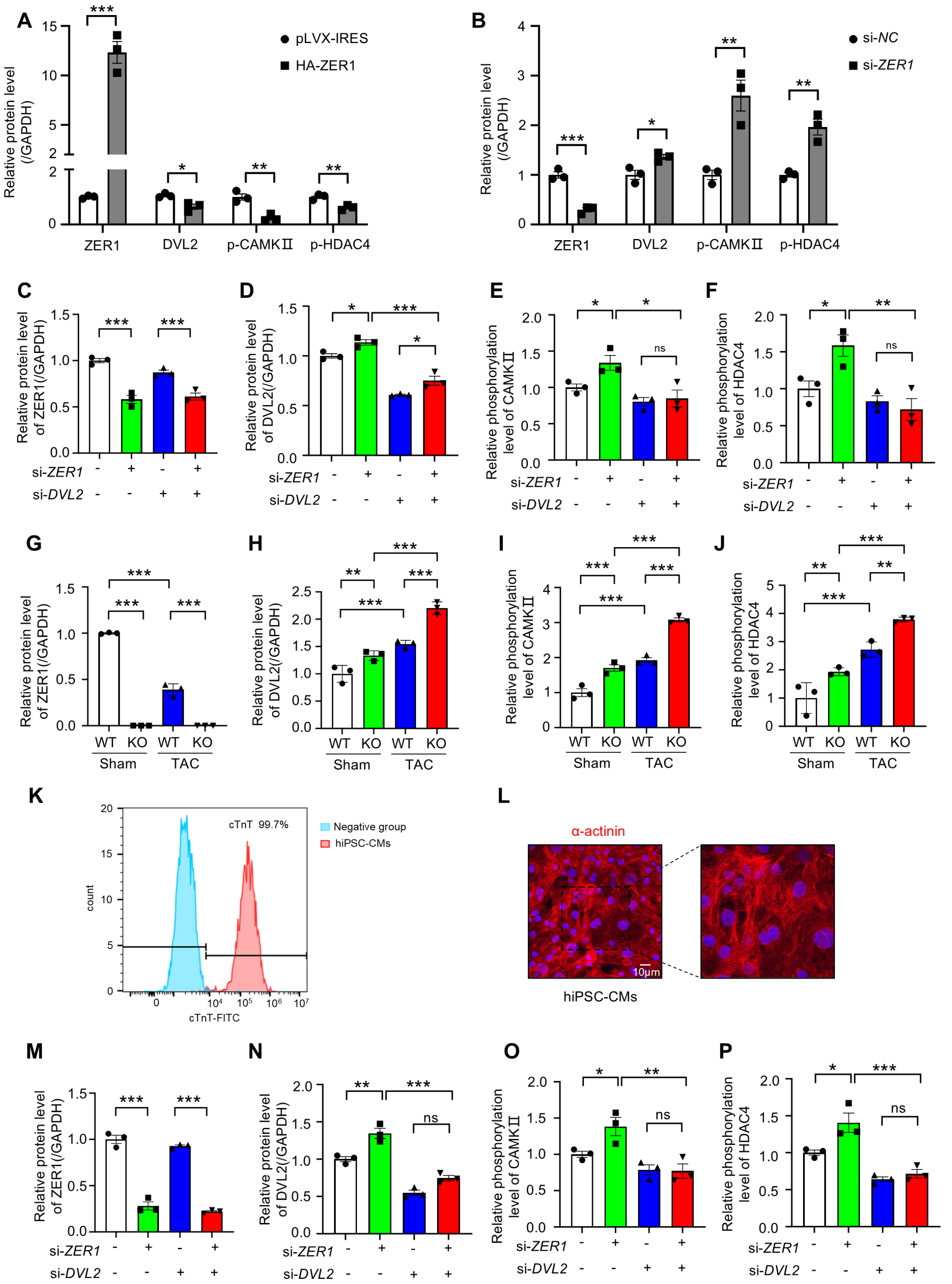

Supplementary Fig. 9. Quantification of DVL2-CaMKII-HDAC4 signaling downstream of ZER1.

(A-F) Quantification of ZER1, DVL2, p-CaMKII (Thr287), and p-HDAC4 (Ser246) in HEK293T cells following HA-ZER1 overexpression, *ZER1* knockdown, or combined *ZER1* and *DVL2* knockdown. **(G-J)** Quantification of ZER1, DVL2, p-CaMKII (Thr287), and p-HDAC4 (Ser246) in WT and *Zer1* KO mouse hearts after TAC. **(K)** Flow cytometry analysis of cTnT-positive hiPSC-derived cardiomyocytes. **(L)** Immunofluorescence staining of α-actinin in hiPSC-derived cardiomyocytes. Nuclei, DAPI. Scale bar, 10 μm. **(M-P)** Quantification of ZER1, DVL2, p-CaMKII (Thr287), and p-HDAC4 (Ser246) in hiPSC-derived cardiomyocytes following *ZER1* knockdown, *DVL2* knockdown, or combined knockdown. Data are presented as mean ± SEM. Statistical analyses were performed using unpaired two-tailed Student's t test or Mann-Whitney U test for two-group comparisons, and two-way ANOVA followed by Holm-Šídák's post hoc test for multi-group comparisons. ns, not significant; *$P < 0.05$, **$P < 0.01$, and ***$P < 0.001$.

Supplemental Figure 10

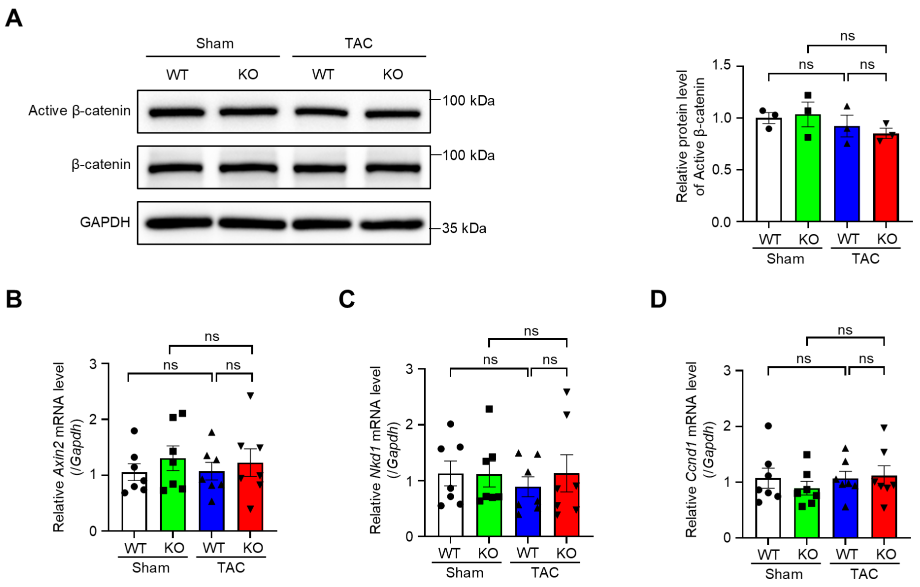

Supplementary Fig. 10. Canonical WNT pathway markers in cardiomyocyte-specific *Zer1*-deficient hearts. (A) Immunoblot analysis and quantification of active β-catenin and total β-catenin protein levels in WT and *Zer1*-KO mouse hearts after sham or TAC. **(B-D)** RT-qPCR analysis of *Axin2*, *Nkd1*, and *Ccnd1* mRNA levels in mouse hearts after sham or TAC; values were normalized to *Gapdh*. Data are presented as mean ± SEM. Statistical analyses were performed using two-way ANOVA followed by Holm-Šídák's post hoc test. ns, not significant.

Supplemental Figure 11

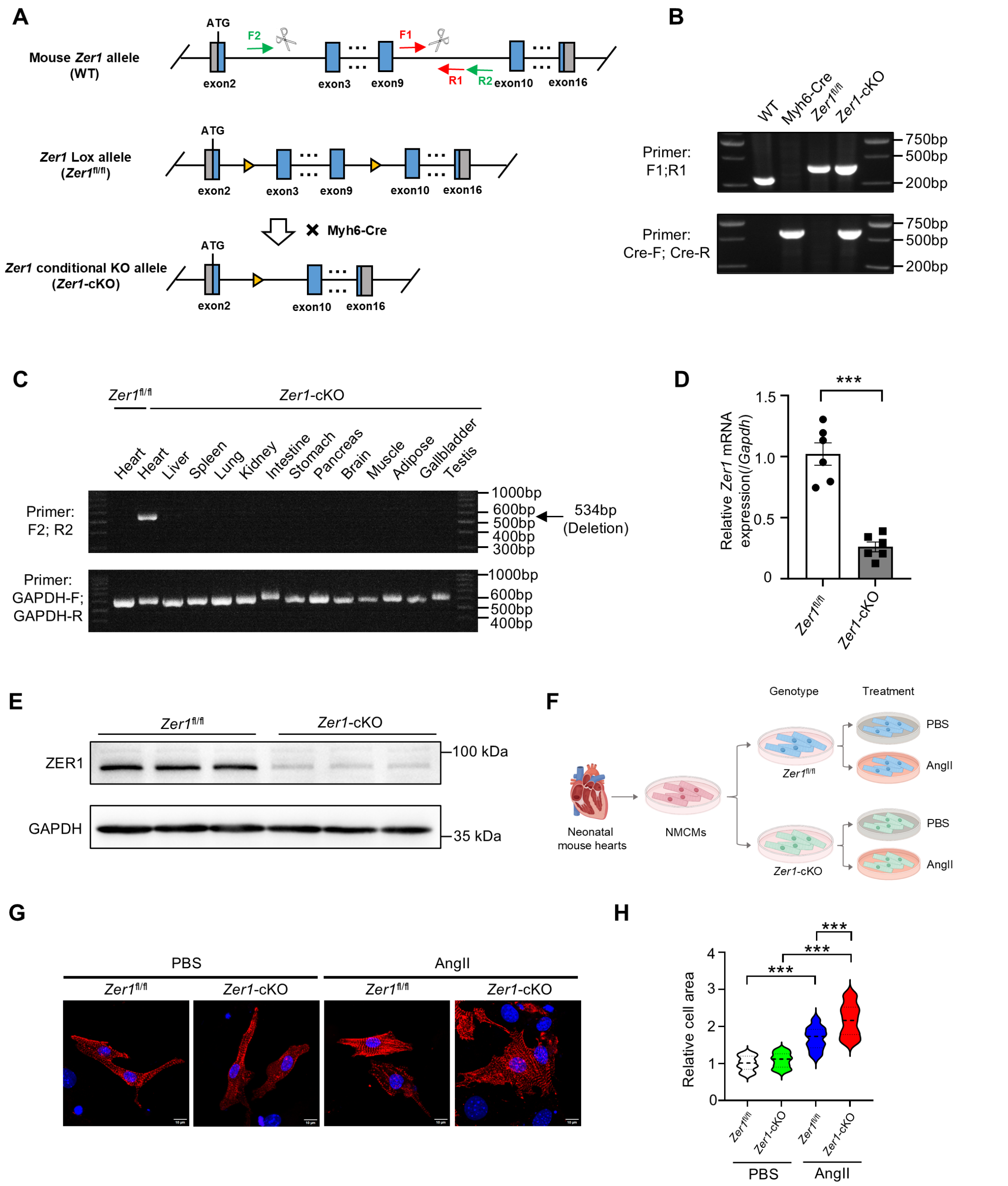

Supplementary Fig. 11. Validation of cardiomyocyte-specific *Zer1* deletion and NMCM hypertrophic growth. (A) Strategy for generating the *Zer1* conditional allele and cardiomyocyte-specific *Zer1* deletion driven by Myh6-Cre. **(B)** PCR genotyping of Myh6-Cre, *Zer1*$^{fl/fl}$, and *Zer1*-cKO mice. **(C)** Tissue-specific knockout validation showing deletion in hearts from *Zer1*-cKO mice. **(D)** RT-qPCR analysis of *Zer1* mRNA

in hearts from *Zer1*$^{fl/fl}$ and *Zer1*-cKO mice. **(E)** Immunoblot analysis of ZER1 protein in hearts from *Zer1*$^{fl/fl}$ and *Zer1*-cKO mice. **(F)** Schematic of NMCM isolation from *Zer1*$^{fl/fl}$ and *Zer1*-cKO mice followed by PBS or AngII treatment. **(G)** Representative α-actinin immunofluorescence images of NMCMs treated with PBS or AngII. Scale bar, 20 μm. **(H)** Quantification of NMCM surface area in (G) (50 cells per group). Data are presented as mean ± SEM. Statistical analyses were performed using unpaired two-tailed Student's t test for two-group comparisons and two-way ANOVA followed by Holm-Šídák's post hoc test for multi-group comparisons. ***$P < 0.001$.

Supplemental Figure 12

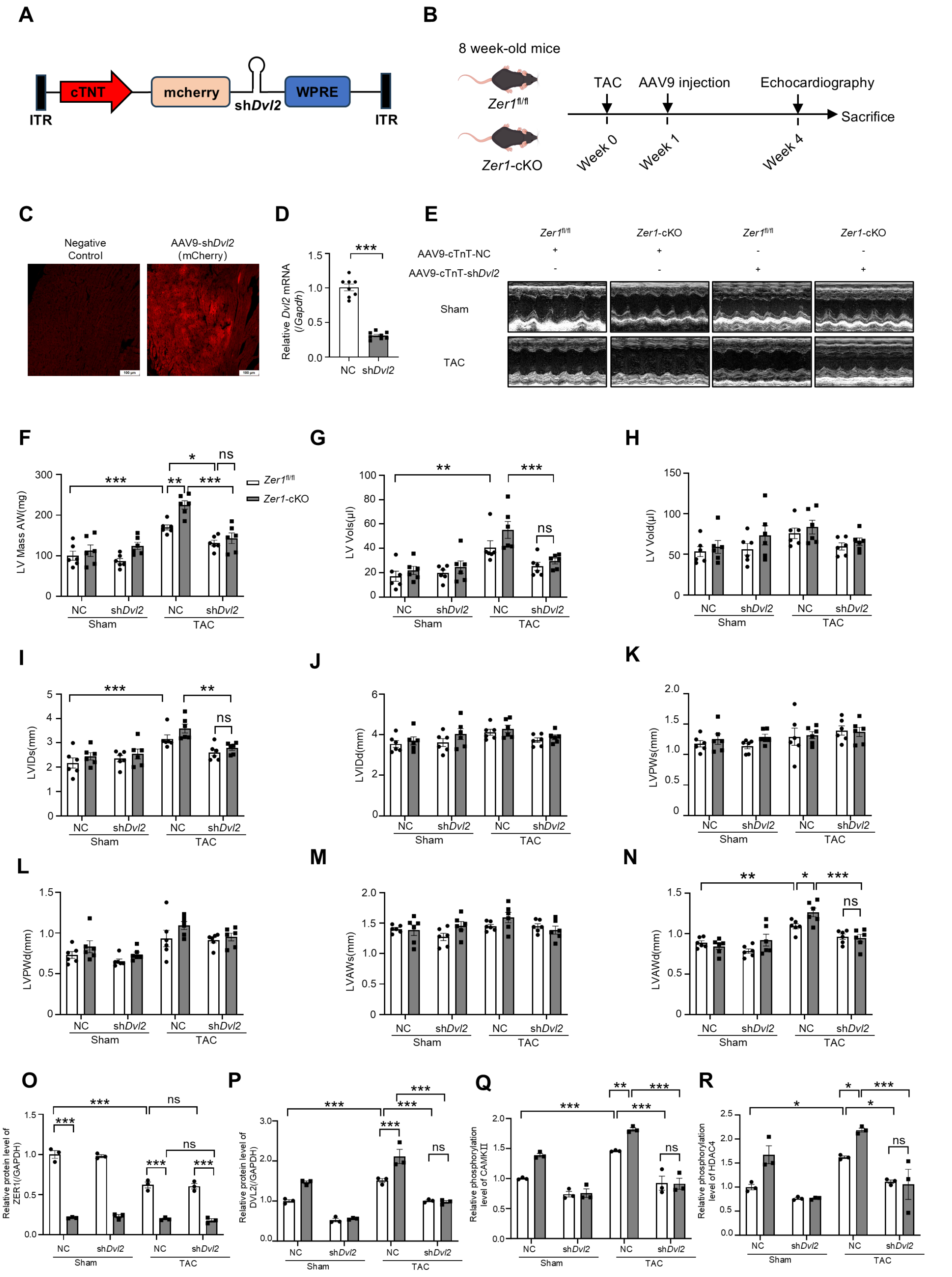

Supplementary Fig. 12. Cardiomyocyte-specific knockdown of *Dvl2* using AAV9-cTnT-sh*Dvl2*. (A) AAV9-cTnT-sh*Dvl2* vector design. **(B)** Experimental timeline for TAC, AAV9 delivery, echocardiography, and tissue collection in *Zer1*$^{fl/fl}$ and *Zer1*-cKO

mice. **(C)** Representative mCherry fluorescence showing cardiac transduction by AAV9-cTnT-sh*Dvl2*. **(D)** RT-qPCR validation of *Dvl2* mRNA knockdown in AAV9-cTnT-sh*Dvl2*-treated hearts. **(E)** Representative M-mode echocardiographic images from mice with the indicated genotypes and AAV9 treatments after sham or TAC. **(F-N)** Echocardiographic parameters in *Zer1*$^{fl/fl}$ and *Zer1*-cKO mice treated with AAV9-cTnT-NC or AAV9-cTnT-sh*Dvl2* after sham or TAC. **(O-R)** Quantification of ZER1, DVL2, p-CaMKII (Thr287), and p-HDAC4 (Ser246) in cardiac lysates from the indicated groups. Data are presented as mean ± SEM. Statistical analyses were performed using two-way ANOVA followed by Holm-Šídák's post hoc test. ns, not significant; **P* < 0.05, ***P* < 0.01, and ****P* < 0.001.

Supplemental Figure 13

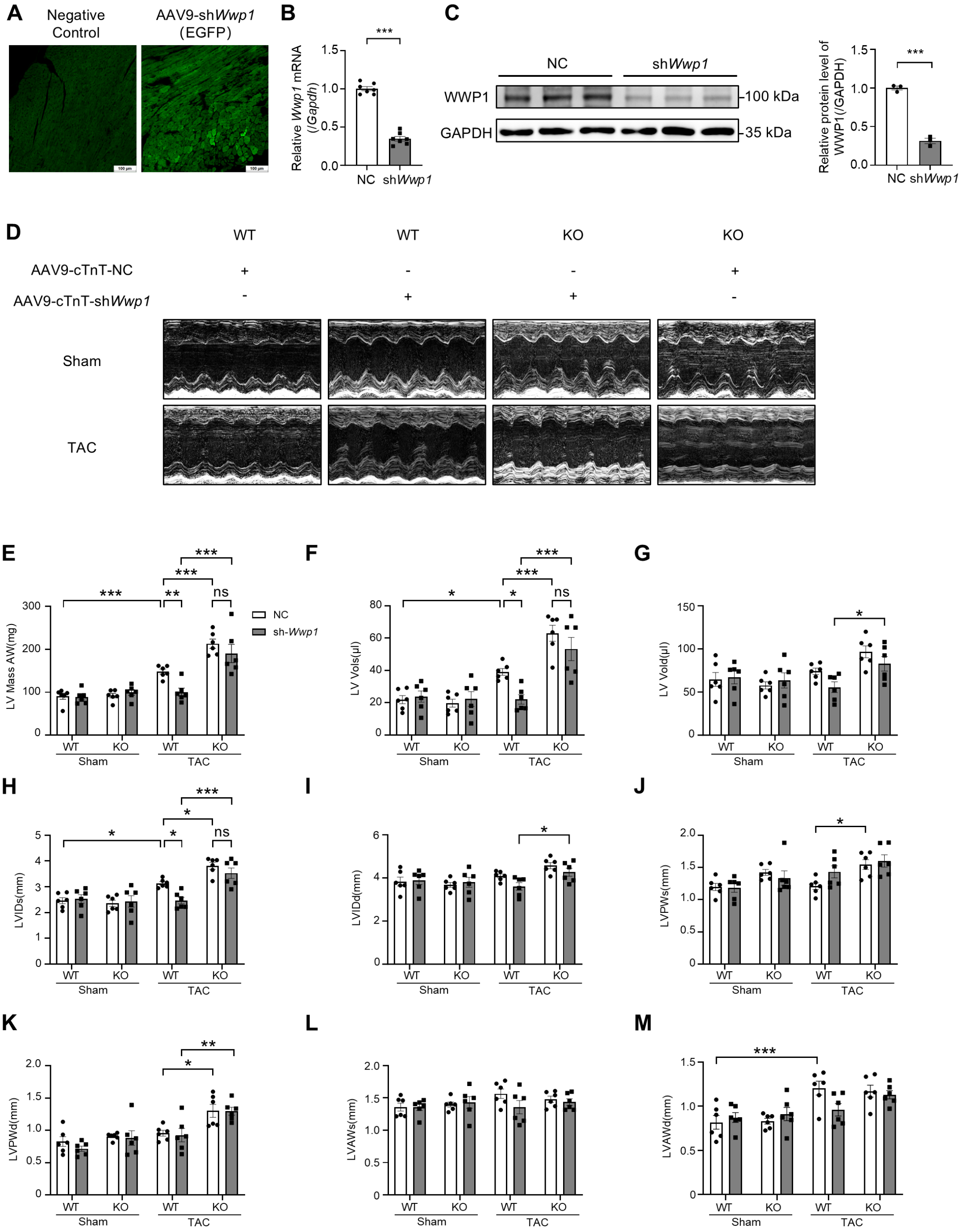

Supplemental Figure 13. Validation and echocardiographic assessment of AAV9-cTnT-sh*Wwp1*. (A) Cardiac AAV9 transduction assessed by EGFP fluorescence. **(B)** RT-qPCR analysis of *Wwp1* mRNA in hearts treated with AAV9-cTnT-NC or AAV9-cTnT-sh*Wwp1*. **(C)** Immunoblot analysis and quantification of WWP1 protein in hearts

treated with AAV9-cTnT-NC or AAV9-cTnT-sh*Wwp1*. **(D)** Representative M-mode echocardiographic images from WT and *Zer1* KO mice treated with AAV9-cTnT-NC or AAV9-cTnT-sh*Wwp1* after sham or TAC. **(E-M)** Echocardiographic parameters in the indicated groups. Data are presented as mean ± SEM. Statistical analyses were performed using unpaired two-tailed Student's t test for two-group comparisons and two-way ANOVA followed by Holm-Šídák's post hoc test for multi-group comparisons. ns, not significant; *$P < 0.05$, **$P < 0.01$, and ***$P < 0.001$.

Supplemental Figure 14

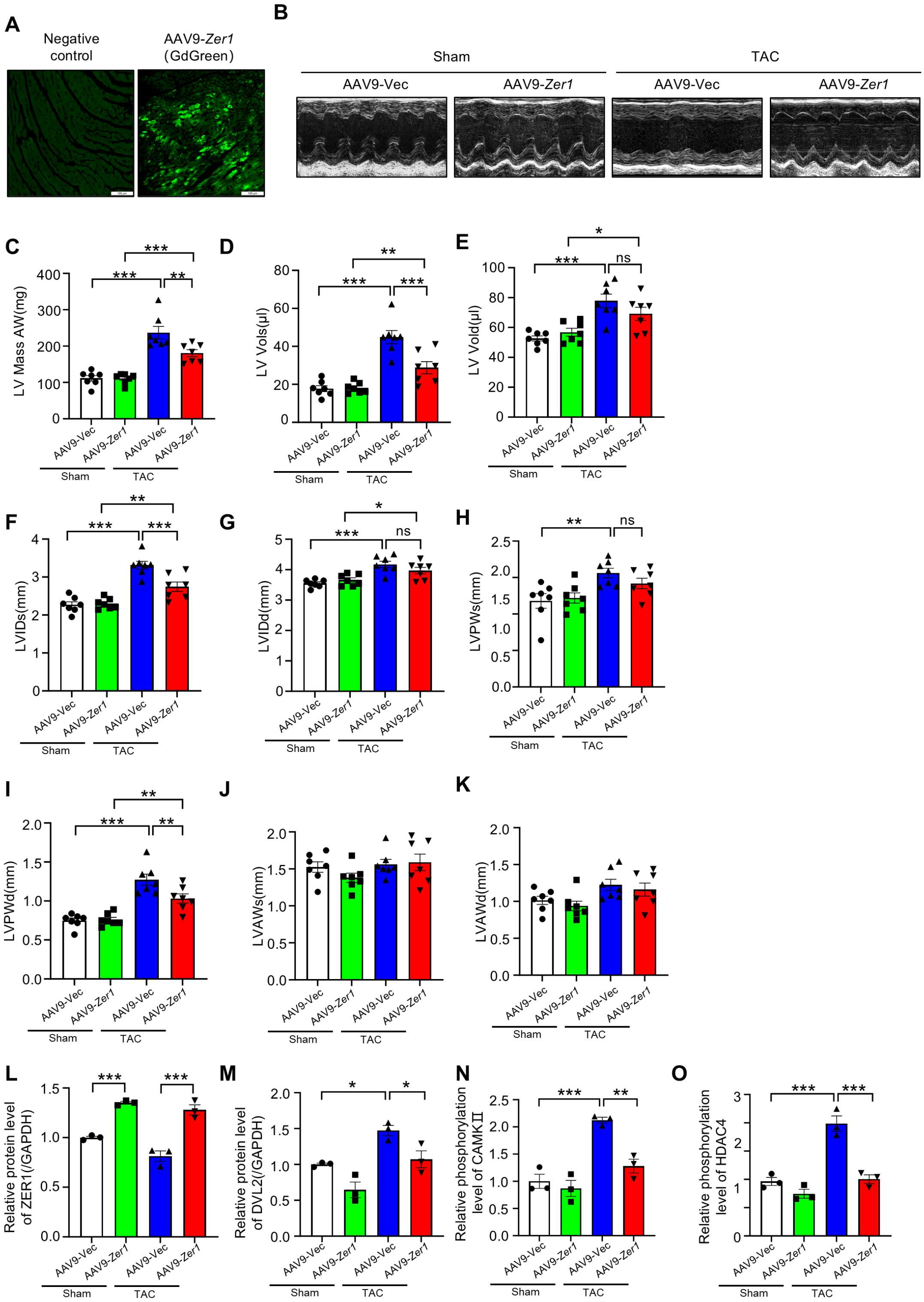

Supplementary Fig. 14. Cardiac transduction and echocardiographic assessment after therapeutic ZER1 expression. (A) Representative GdGreen fluorescence images confirming cardiac AAV9 transduction. **(B)** Representative M-mode echocardiographic

images from mice treated with AAV9-cTnT-*ZER1* or AAV9-cTnT-Vector after sham or TAC. **(C-K)** Echocardiographic parameters in mice treated with AAV9-cTnT-*ZER1* or AAV9-cTnT-Vector after sham or TAC. **(L-O)** Quantification of ZER1 and DVL2 protein abundance and CaMKII/HDAC4 phosphorylation in cardiac lysates from the indicated groups. Data are presented as mean ± SEM. Statistical analyses were performed using two-way ANOVA followed by Holm-Šídák's post hoc test. ns, not significant; *$P < 0.05$, **$P < 0.01$, and ***$P < 0.001$.

Supplemental Table 1. Patient Characteristics

Sample ID	Sex	Age (yr)	Diagnosis	LVEF (%)
NF1	M	39	Brain death (Traffic Accident)	NA
NF2	F	50	Brain death (Traffic Accident)	NA
NF3	M	29	Brain death (Traffic Accident)	NA
NF4	F	47	Brain death (Traffic Accident)	NA
HF1	M	65	DCM	15
HF2	M	75	DCM	18
HF3	M	53	DCM	24
HF4	M	69	DCM	18

DCM, dilated cardiomyopathy; LVEF, left ventricular ejection fraction; NF, non-failing hearts; HF, failing hearts.

Supplemental Table 2. Full prioritized interactome list with all filtering annotations (n=53)

Gene_symbol	UniProt_ID	log2FC_ZER1_vs_IgG	adj_p_value	Nterm_2aa	Met_cleavage_predicted	Nt1_in_GASTC	Tier
Mccc2	Q3ULD5	5.484859922	0.00617697	MW	FALSE	FALSE	2
Camk2d	Q6PHZ2	5.183670999	0.00821957	MA	TRUE	TRUE	1
Atp2a2	O55143	5.021522853	0.009553259	ME	FALSE	FALSE	2
Cox5a	P12787	5.021522853	0.009553259	ML	FALSE	FALSE	2
Habp2	Q8K0D2	4.989812257	0.032627281	MF	FALSE	FALSE	2
Dvl2	Q60838	4.984883266	0.000874726	MA	TRUE	TRUE	1
Ezr	P26040	4.342184918	0.014386424	MP	TRUE	FALSE	1
Zer1	Q80ZJ6	4.270951815	4.01371E-07	MA	TRUE	TRUE	2
Fhl2	O70433	4.21204496	0.008329759	MT	TRUE	TRUE	1
Opa1	P58281	4.133389529	0.000935678	MW	FALSE	FALSE	2
Eloc	P83940	4.008313644	3.1988E-05	MD	FALSE	FALSE	2
Sorbs1	Q62417	3.628572921	0.045348923	MS	TRUE	TRUE	1
Ctnna3	Q65CL1	3.628119902	0.000280581	MS	TRUE	TRUE	1
Cbr4	Q91VT4	3.461510982	0.000782257	MD	FALSE	FALSE	2
mt-Co3	P00416	3.332057452	4.66916E-05	MT	TRUE	TRUE	2
Ywhaq	P68254	3.28419599	0.006519367	ME	FALSE	FALSE	2
Cd5l	Q9QWK4	3.162223532	0.044685585	MA	TRUE	TRUE	1
Cul2	Q9D4H8	3.057076664	0.000847489	MS	TRUE	TRUE	2
Dstn	Q9R0P5	3.009069745	1.68277E-06	MA	TRUE	TRUE	1
Hsdl2	Q2TPA8	2.812234845	9.53312E-05	ML	FALSE	FALSE	2
Dglucy	Q8BH86	2.805111983	0.000922415	MT	TRUE	TRUE	2
Rack1	P68040	2.766134257	0.028019407	MT	TRUE	TRUE	1
Dsc1	P55849	2.733796943	0.01178575	MA	TRUE	TRUE	2
Rps18	P62270	2.587210702	0.025075483	MS	TRUE	TRUE	2
Serpinb6	Q60854	2.584613241	0.043469816	MD	FALSE	FALSE	2
Col14a1	Q80X19	2.403099159	0.002452493	MM	FALSE	FALSE	2
Cox16	Q9CR63	2.073527443	0.000764722	MI	FALSE	FALSE	2
Nid1	P10493	2.01825475	0.000554255	ML	FALSE	FALSE	2
Ndufb10	Q9DCS9	1.996919416	0.005628836	MP	TRUE	FALSE	2
Ppp1ca	P62137	1.766217088	0.000957679	MS	TRUE	TRUE	1
Lgals1	P16045	1.676197606	0.030034483	MA	TRUE	TRUE	1
Eno1	P17182	1.558844706	0.00018037	MS	TRUE	TRUE	1
Ndufb7	Q9CR61	1.558089757	0.001686463	MG	TRUE	TRUE	2
Cav2	Q9WVC3	1.522542223	0.006901169	MG	TRUE	TRUE	2
Selenbp1	P17563	1.51520028	0.011132419	MA	TRUE	TRUE	2
Pls3	Q99K51	1.4875698	0.001090739	MD	FALSE	FALSE	2
Gpd1	P13707	1.442856976	5.60627E-05	MA	TRUE	TRUE	2

Rab1A	P62821	1.395255701	9.22118E-05	MS	TRUE	TRUE	2
Adh5	P28474	1.39323663	0.008919732	MA	TRUE	TRUE	2
Pgam1	Q9DBJ1	1.391169202	0.001802798	MA	TRUE	TRUE	2
Bcl2l13	P59017	1.388219144	0.034793469	MA	TRUE	TRUE	1
Myl7	Q9QVP4	1.355175671	0.005645878	MA	TRUE	TRUE	1
Hadh	Q61425	1.332780724	0.007254843	MA	TRUE	TRUE	2
Ptgr3	Q8BGC4	1.287942288	0.02500423	ML	FALSE	FALSE	2
Hpx	Q91X72	1.266983046	0.002125265	MA	TRUE	TRUE	2
Aldh1b1	Q9CZS1	1.265983356	0.00147977	ML	FALSE	FALSE	2
Hvm02	P01746	1.239679787	0.028761781	MG	TRUE	TRUE	2
Mpi	Q924M7	1.129110972	0.005316123	MA	TRUE	TRUE	2
Krt16	Q9Z2K1	1.124886965	0.008324036	MA	TRUE	TRUE	1
Tmem263	Q9DAM7	1.107676668	0.003337213	MN	FALSE	FALSE	2
Hvm17	P01786	1.104546794	0.016750909	EV	TRUE	FALSE	2
Cep250	Q60952	1.090309451	0.013478848	ME	FALSE	FALSE	2
Ube2l3	P68037	1.049445733	0.011351472	MA	TRUE	TRUE	2

Supplemental Table 3. Final Tier 1 candidate list ranked by IP-MS enrichment (n=15)

Rank_within_Tier1	Gene_symbol	log2FC_ZER1_vs_IgG	adj_p_value	Mature Nt1	Tier1_GO_BP_hit
1	Camk2d	5.183670999	0.00821957	A	regulation of cardiac muscle hypertrophy and cardiac muscle cell apoptotic process
2	Dvl2	4.984883266	0.000874726	A	heart looping | heart morphogenesis | cytoskeleton | heart development
3	Ezr	4.342184918	0.014386424	P	actin filament | actin cytoskeleton
4	Fhl2	4.21204496	0.008329759	T	heart trabecula formation | regulation of apoptotic process
5	Sorbs1	3.628572921	0.045348923	S	cytoskeleton
6	Ctnna3	3.628119902	0.000280581	S	cytoskeleton | actin filament binding | regulation of heart rate
7	Cd5l	3.162223532	0.044685585	A	apoptotic process
8	Dstn	3.009069745	1.68277E-06	A	actin cytoskeleton | actin filament binding | actin filament depolymerization
9	Rack1	2.766134257	0.028019407	T	apoptotic process
10	Ppp1ca	1.766217088	0.000957679	S	regulation of extrinsic apoptotic signaling pathway
11	Lgals1	1.676197606	0.030034483	A	collagen-containing extracellular matrix | apoptotic process
12	Eno1	1.558844706	0.00018037	S	regulation of hypoxia-induced intrinsic apoptotic signaling pathway
13	Bcl2l13	1.388219144	0.034793469	A	apoptotic process | regulation of apoptotic process
14	Myl7	1.355175671	0.005645878	A	myofibril | cardiac muscle tissue development | heart contraction
15	Krt16	1.124886965	0.008324036	A	cytoskeleton | intermediate filament cytoskeleton organization
